# Synthesis and *in vivo* characterization of ^18^F-labeled difluoroboron-curcumin derivative for β-amyloid plaque imaging

**DOI:** 10.1038/s41598-019-43257-9

**Published:** 2019-05-01

**Authors:** Hyunjung Kim, Young Hoon Im, Jinhee Ahn, Jehoon Yang, Joon Young Choi, Kyung-Han Lee, Byung-Tae Kim, Yearn Seong Choe

**Affiliations:** 1 Department of Health Sciences and Technology, SAIHST, Sungkyunkwan University, Seoul, 06351 Korea; 2 Department of Nuclear Medicine, Samsung Medical Center, Sungkyunkwan University School of Medicine, Seoul, 06351 Korea; 3 Laboratory Animal Research Center, Samsung Medical Center, Seoul, 06351 Korea

**Keywords:** Chemical modification, Natural products

## Abstract

Positron emission tomography imaging of β-amyloid (Aβ) plaques has proven useful in the diagnosis of Alzheimer’s disease. A previous study from our group showed that 4′-*O*-[^18^F]fluoropropylcurcumin has poor brain permeability, which is thought to be due to its rapid metabolism. In this study, we synthesized difluoroboron complexes of fluorine-substituted curcumin derivatives (**1**–**4**) and selected one of them based on the *in vitro* binding assays. The selected ligand **2** was found to distinctively stain Aβ plaques in APP/PS1 transgenic mouse brain sections. Radioligand [^18^F]**2** was synthesized via a two-step reaction consisting of [^18^F]fluorination and subsequent aldol condensation. Biodistribution and metabolism studies indicated that radioligand [^18^F]**2** was converted to polar radioactive products and trapped in the normal mouse brain. In contrast, optical images of mice acquired after injection of **2** showed moderate fluorescence signal intensity in the mouse brain at 2 min with a decrease in the signal within 30 min. In the *ex vivo* optical images, the fluorescence signals in major tissues disappeared within 30 min. Taken together, these results suggest that [^18^F]**2** may be converted to polar ^18^F-labeled blue-shifted fluorescent products. Further structural modifications are thus needed to render the radioligand metabolically stable.

## Introduction

**Figure 1:**
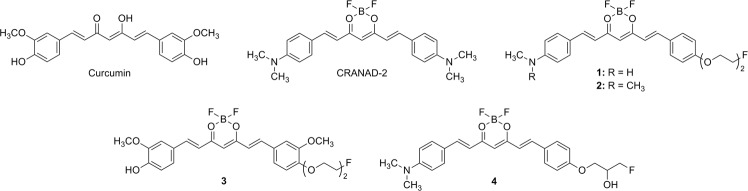
Chemical structures of curcumin, CRANAD-2, and ligands (**1**–**4**).

Alzheimer’s disease (AD) is a neurodegenerative disorder that is characterized by the accumulation of β-amyloid (Aβ) plaques and neurofibrillary tangles in human brain. Positron emission tomography (PET) radiopharmaceuticals for Aβ plaque imaging have been approved by Food and Drug Administration for the diagnosis of AD. These radiopharmaceuticals include Amyvid, Neuraceq, and Vizamyl. Curcumin, which is a major component of the curry spice turmeric, is also known to have anti-amyloidogenic activity^1^. Therefore, curcumin has been a focus of recent interest. One study found that curcumin inhibited Aβ fibril formation *in vitro* and labeled amyloid plaques when it was incubated with Tg2576 mouse brain and AD brain sections. In addition, a diet rich in curcumin lowered amyloid plaque burden in transgenic mice^2^. In this context, we developed 4′-*O*-fluoropropylcurcumin, which has an *in vitro* binding affinity for Aβ aggregates that is much higher than that of curcumin^3^. However, its radiolabeled form, 4′-*O*-[^18^F]fluoropropylcurcumin has poor brain permeability that is likely due to its rapid metabolism, like curcumin^3^. The other ^18^F-labeled curcumin derivatives also demonstrated low brain uptake^4,5^. More recently, Ran *et al*. reported synthesis of difluoroboron-curcumin derivatives and their application to near-infrared (NIR) imaging of Aβ plaques in transgenic mice^6^. The difluoroboron-curcumin was initially reported to have inhibitory activity on HIV-1 protease that is more potent than that of curcumin (IC_50_ = 24 μM vs. 100 μM)^7^. In addition, the complexation of a difluoroboron group to the 3,5-diketone moiety of curcumin caused a red shift in the absorption. Similarly, rosocyanine and rubrocurcumin, which are produced from the reaction of curcumin and boric acid under different conditions, are red-colored solids whose absorptions are red-shifted from the yellow color of curcumin^8,9^. Ran *et al*. reported that the difluoroboron-curcumin showed significant red and Stokes shifts compared to those of curcumin (excitation, 540 nm vs. 510 nm; emission, 640 nm vs. 560 nm in methanol). This characteristic of difluoroboron-curcumin enables optical imaging^6^. CRANAD-2 is a difluoroboron complex of curcumin substituted with dimethylamino groups at both phenol rings (Fig. 1). This ligand exhibited high binding affinity for Aβ(1–40) aggregates (K_d_ = 38 nM), desirable lipophilicity (log P = 3), and stability in the serum. Furthermore, CRANAD-2 caused a large increase in the fluorescence intensity and quantum yield, as well as a 90-nm blue shift when binding to the Aβ aggregates^6^. Intravenous injection of this ligand to Tg2576 mice produced a significantly higher fluorescence signal in the brain than did that of age-matched wild-type mice^6^. A series of CRANAD compounds, in which phenyl rings are substituted with diverse groups, were synthesized. One of these compounds demonstrated strong NIR signals in 4-month-old transgenic mouse brains where soluble Aβ species are most likely to exist^10^. NIR imaging may be useful for screening candidate ligands because it can be obtained in real time; however, it is limited in its depth penetration. In contrast, PET imaging provides sensitive and quantitative information using tracer doses of radioligand. PET imaging agents can also be readily translated to the clinical setting.

In this study, therefore, we synthesized difluoroboron-curcumin derivatives substituted with fluorine-containing groups at one of the phenyl rings (Fig. 1). Of the derivatives, we selected the most appropriate ligand (**2**) based on its *in vitro* binding affinity for Aβ aggregates. We then synthesized ^18^F-labeled ligand ([^18^F]**2**), and evaluated the radioligand as an Aβ plaque imaging agent.

## Results

### Synthesis of non-radioactive ligands

**Figure 2:**
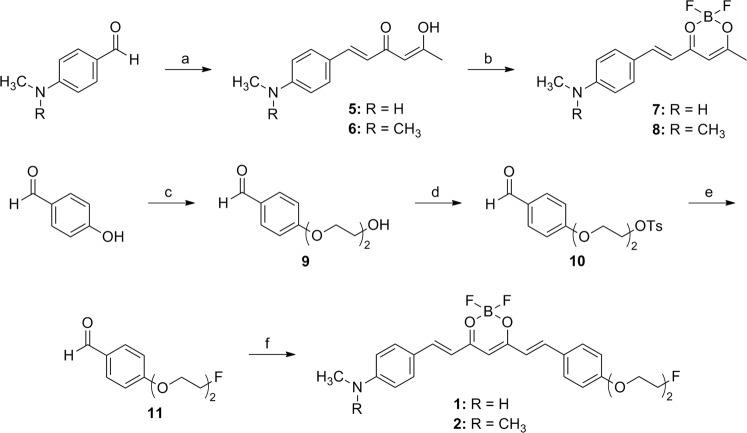
Synthesis of **1** and **2**. Reagents and conditions: (**a**) 2,4-pentanedione, B_2_O_3_, ethyl acetate, 80 °C, 0.5 h, *n*-BuNH_2_, 100 °C, 1 h, 0.4 N HCl, 50 °C, 0.5 h; (**b**) BF_3_·Et_2_O, CH_2_Cl_2_, rt, 3 h; (**c**) 2-(2-chloroethoxy)ethanol, K_2_CO_3_, DMF, 100 °C, overnight; (**d**) TsCl, Et_3_N, CH_2_Cl_2_, rt, overnight; (**e**) CsF, *t*-BuOH, 100 °C, overnight; (**f**) **7** or **8**, piperidine, ethyl acetate, 110 °C, 20 min.

**Figure 3:**
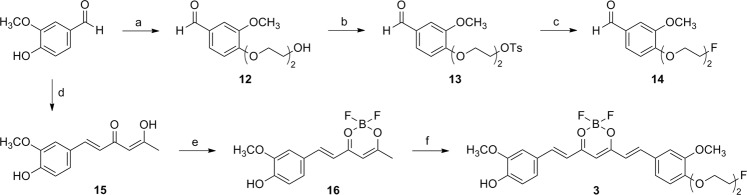
Synthesis of **3**. Reagents and conditions: (**a**) 2-(2-chloroethoxy)ethanol, DMF, K_2_CO_3_, 100 °C, overnight; (**b**) TsCl, CH_2_Cl_2_, Et_3_N, rt, overnight; (**c**) CsF, *t*-BuOH, 100 °C, overnight; (**d**) 2,4-pentanedione, B_2_O_3_, (*n*-BuO)_3_B, ethyl acetate, 80 °C, 30 min, *n*-BuNH_2_, 100 °C, 1 h, 0.4 N HCl, 50 °C, 30 min; (**e**) BF_3_·Et_2_O, CH_2_Cl_2_, rt, 2 h; (**f**) **14**, (*n*-BuO)_3_B, piperidine, ethyl acetate, 110 °C, 20 min.

**Figure 4:**
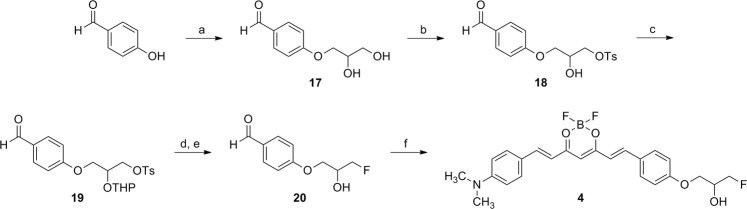
Synthesis of **4**. Reagents and conditions: (**a**) (±)-3-chloro-1,2-propanediol, NaOH, EtOH, 100 °C, overnight; (**b**) TsCl, CH_2_Cl_2_, Et_3_N, rt, overnight; (**c**) 3,4-dihydro-2*H*-pyran, pyridinium *p*-toluenesulfonate, CH_2_Cl_2_, reflux, 4 h; (**d**) CsF, *t*-BuOH, 110 °C, overnight; (**e**) 1 N HCl, CH_3_CN, 100 °C, 5 min; (**f**) **8**, piperidine, ethyl acetate, 110 °C, 20 min.

Ligands **1** and **2** were synthesized from condensation reactions of difluoroboron compounds **7** and **8** with 4-(2-(2-fluoroethoxy)ethoxy)benzaldehyde (**11**) in the presence of piperidine, respectively (Fig. 2). Compounds **7** and **8** were prepared by the aldol condensation reaction of 2,4-pentanedione and benzaldehyde derivatives, followed by difluoroboron complex formation^3,6,11^. Compound **11** was prepared from 4-hydroxybenzaldehyde in three steps in high yield; the final fluorination step was carried out by reacting **10** with CsF in *t*-BuOH, which provided **11** in higher yield than in acetonitrile^12^. Ligand **3** was synthesized from condensation reaction of **14** with difluoroboron compound **16** in the presence of piperidine (Fig. 3). Compounds **14** and **16** were prepared from vanillin in 3 and 2 steps, respectively. Ligand **4** was synthesized from condensation reaction of **8** with 4-(3-fluoro-2-hydroxypropoxy)benzaldehyde (**20**) in the presence of piperidine (Fig. 4). Compound **20** was prepared from 4-hydroxybenzaldehyde in five steps; reaction of 4-hydroxybenzaldehyde and (±)-3-chloro-1,2-propanediol, tosylation of the 3-hydroxy group, protection of the 2-hydroxy group, fluorination of the tosylate group, and then deprotection of the THP group. Ligands **1**, **3**, and **4** were not completely pure after flash column chromatography. This result is likely due to the presence of the monomethylamino group or hydroxy group in those ligands. Therefore, all the ligands were purified by HPLC after flash column chromatography.

### Radiochemical synthesis

**Figure 5:**
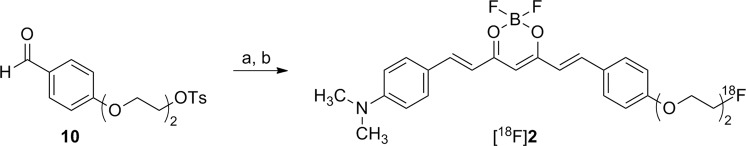
Synthesis of [^18^F]**2**. Reagents and conditions: (**a**) *n*-Bu_4_[^18^F]F, CH_3_CN, 110 °C, 10 min; (**b**) **8**, *n*-butylamine, ethyl acetate, 110 °C, 20 min.

Radioligand [^18^F]**2** was synthesized by [^18^F]fluorination of **10**, followed by aldol condensation with **8** (Fig. 5). A small amount of the precursor (1.4 μmol) was used for radiolabeling to increase the molar activity of the radioligand. Displacement of the base from piperidine to *n*-butylamine and reduction in amount of the base increased the yield of the aldol condensation reaction from 9% to 37%^11^. The following HPLC purification gave [^18^F]**2** in overall 17–22% decay-corrected radiochemical yield with a molar activity of 39–46.8 GBq/μmol.

### Excitation and emission spectra

Ligand **2** showed a red-shifted emission and a larger Stokes shift (excitation 550 nm, emission 650) compared to those of curcumin (excitation 510 nm, emission 560) (Supplementary Fig. S15)^6^. CRANAD-2 displayed far more red-shifted emission (760 nm) than did ligand **2** because of the presence of dimethylamino groups at both ends (Fig. 1). Despite this, ligand **2** has sufficient red-shifted emission for optical imaging.

### *In vitro* binding assays using Aβ(1–42) aggregates

Binding constants of the ligands for Aβ(1–42) aggregates were determined using the intrinsic fluorescence of the ligands. The binding constant of CRANAD-2 was also measured for comparison. Its K_d_ value measured in this study was 19.40 nM, while the reported value was 38.69 nM using Aβ(1–40) aggregates^6^. The ligand with a dimethylamino group (**2**) exhibited higher binding affinity than did the ligand with a monomethylamino group (**1**) (K_d_ = 19.66 nM vs. 33.82 nM) (Supplementary Fig. S16). Ligand **3** had the lowest binding affinity (K_d_ = 138.5 nM), while ligand **4** was shown to have the highest binding affinity (K_d_ = 15.35 nM). Therefore, most ligands exhibited similar binding affinities to CRANAD-2 for Aβ(1–42) aggregates. Considering the K_d_ values and feasibility of ^18^F-labeling of all the ligands, ligand **2** was selected for further *in vitro* and *in vivo* studies.

### Staining of Aβ plaques in double transgenic mouse brain sections

**Figure 6:**
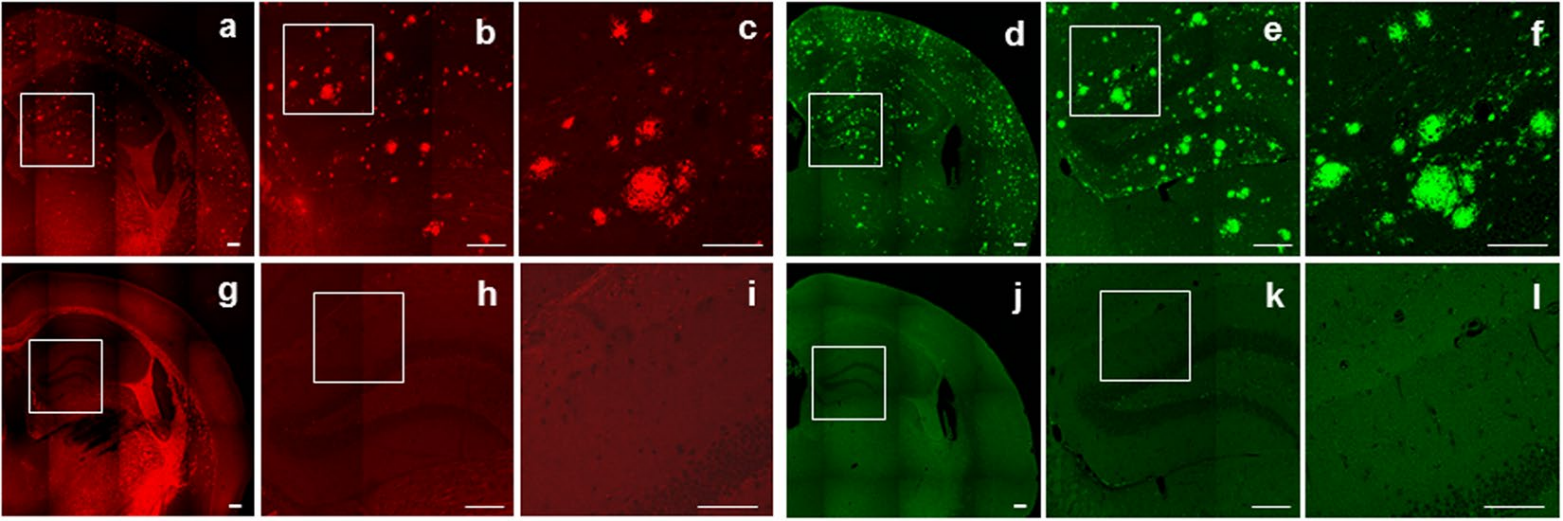
A double transgenic mouse brain section (**a**–**c**) and an age-matched wild-type mouse brain section (**g**–**i**) stained with **2**. The adjacent double transgenic mouse brain section (**d**–**f**) and age-matched wild-type mouse brain section (**j**–**l**) stained with an Aβ-specific antibody. Magnification of the regions in white boxes (**c**,**f**,**i** and **l**). Scale bars: 100 μm (**c**,**f**,**i** and **l**) and 200 μm (the rest).

Fluorescent staining of Aβ plaques by **2** was performed using the brain sections from a double transgenic mouse (Tg APP/PS-1). Localization of the Aβ plaques was confirmed by immunofluorescent staining the adjacent brain sections from the transgenic mouse with an anti-Aβ antibody (Fig. 6). The Aβ plaques in the cerebral cortex and hippocampus regions of the transgenic mouse brain sections were stained with **2** (Fig. 6a–c). The staining pattern was consistent with that observed with an Aβ-specific antibody (Fig. 6d–f). In contrast, there was no notable positive staining by either **2** or anti-Aβ antibody in wild-type mouse brain sections (Fig. 6g–l).

### Partition coefficient measurement

Partition coefficient of [^18^F]**2** was measured using a mixture of 1-octanol and water, and its log P value was 2.58 ± 0.04. This result demonstrates that the radioligand can cross the blood-brain barrier. Difluoroboron complex formation lowered the lipophilicity of the curcumin derivative based on TLC analysis.

### *In vitro* stability study

**Figure 7:**
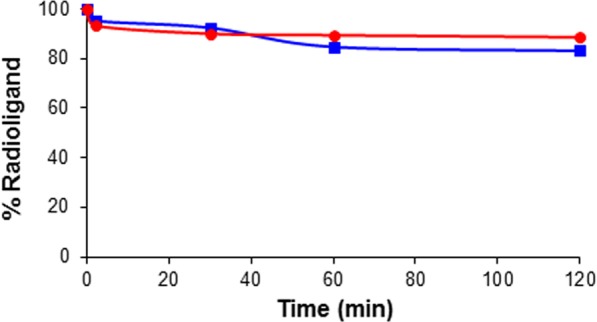
*In vitro* stability of [^18^F]**2** in PBS (●) and FBS (■) upon incubation at 37 °C for 120 min followed by analysis using radio-TLC (4:1 ethyl acetate–hexane). Data are presented as % remaining radioligand.

Stability study was performed by incubating [^18^F]**2** in phosphate-buffered saline (PBS) and in fetal bovine serum (FBS) at 37 °C for 120 min. Radio-TLC data showed that [^18^F]**2** remained 89% in PBS and 83% in FBS at the end of the incubation (Fig. 7). These results indicate that radioligand [^18^F]**2** is fairly stable in both PBS and FBS.

### Biodistribution study

Biodistribution study of [^18^F]**2** in ICR mice demonstrated high radioactivity accumulation in the liver (26.92 ± 1.58% ID/g) 2 min after injection, which decreased by 60 min (9.36 ± 0.18% ID/g at 60 min). In contrast, radioactivity uptake in the small intestine increased significantly over time from 0.92 ± 0.13% ID/g at 2 min to 14.92 ± 4.06% ID/g at 60 min (Table 1). Radioactivity uptake in the spleen decreased slightly over time (12.59 ± 2.42% ID/g at 2 min to 10.72 ± 2.45% ID/g at 60 min). The radioligand did not appear to undergo metabolic defluorination because of low level of femur uptake (0.58 ± 0.10% ID/g at 2 min to 1.43 ± 0.14% ID/g at 60 min) (Table 1). However, there was poor brain uptake at 2 min after injection with increased uptake over time, as follows: 0.49 ± 0.02% ID/g at 2 min, 1.02 ± 0.08% ID/g at 30 min, and 1.19 ± 0.06% ID/g at 60 min (Table 1). This result indicates that there is generation and retention of polar radioactive products in the mouse brain.

**Table 1:** Biodistribution of [^18^F]**2** in ICR mice.

Organ	2 min	30 min	60 min
Blood	1.49 ± 0.12	2.50 ± 0.22	3.46 ± 0.81
Heart	2.20 ± 0.57	1.93 ± 0.19	1.87 ± 0.20
Lung	3.56 ± 0.25	2.62 ± 0.29	2.34 ± 0.50
Liver	26.92 ± 1.58	14.21 ± 1.39	9.36 ± 0.18
Spleen	12.59 ± 2.42	11.66 ± 2.70	10.72 ± 2.45
Kidney	3.25 ± 0.48	4.11 ± 0.46	2.58 ± 0.15
Small intestine	0.92 ± 0.13	6.47 ± 1.63	14.92 ± 4.06
Large intestine	0.24 ± 0.05	0.95 ± 0.11	2.16 ± 0.42
Muscle	0.49 ± 0.19	0.97 ± 0.11	0.87 ± 0.05
Femur	0.58 ± 0.10	0.91 ± 0.13	1.43 ± 0.14
Brain	0.49 ± 0.02	1.02 ± 0.08	1.19 ± 0.06

Values (% ID/g) are given as mean ± SD of groups, n = 4 mice.

### Optical imaging

**Figure 8:**
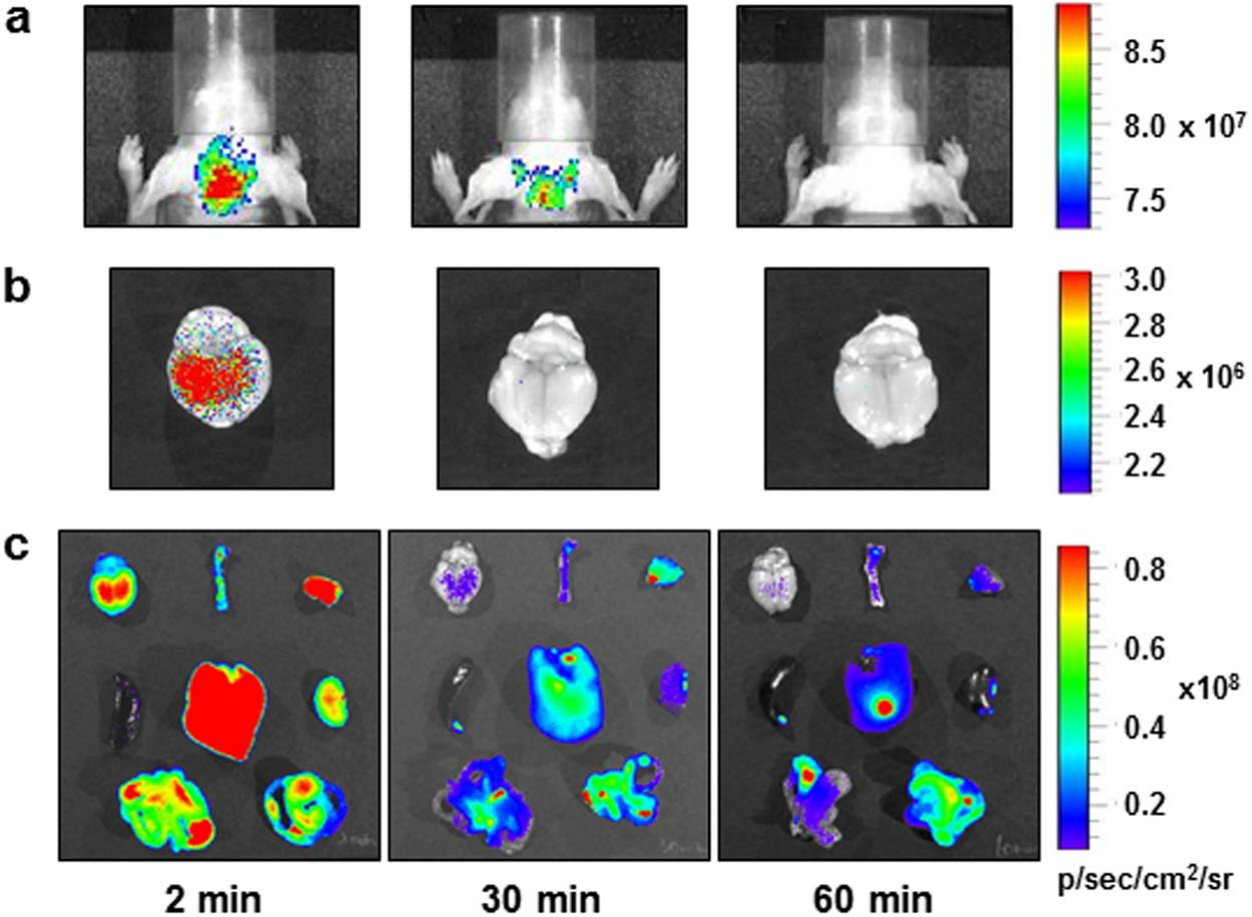
Optical images of Balb/C nude mice obtained at 2, 30, and 60 min after injection of **2** (2 mg/kg); *in vivo* (**a**) and *ex vivo* (**b**) brain images (excitation, 570 nm; emission, 660 nm). *Ex vivo* images of major tissues (**c**): (top row) brain, femur, and muscle; (middle row) spleen, liver, and kidney; (bottom row) small and large intestines.

Optical images of hairless Balb/C nude mice were acquired after injection of **2**. Moderate fluorescence signal intensity was detected in mouse brain at 2 min after injection, which significantly decreased within 30 min (Fig. 8a). *Ex vivo* optical images of the mouse brain was consistent with the result of *in vivo* optical imaging (Fig. 8a–c). *Ex vivo* images also showed moderate to strong fluorescence signals in the muscle, kidney, intestines, and liver at 2 min after injection, all of which decreased to the background level within 30 min (Fig. 8c). In particular, there was rapid disappearance of the fluorescence signals from the organs of elimination. Considering the log P value of **2**, this ligand would have not been eliminated within 30 min after injection, suggesting metabolism/degradation of **2**.

### Metabolism study

**Figure 9:**
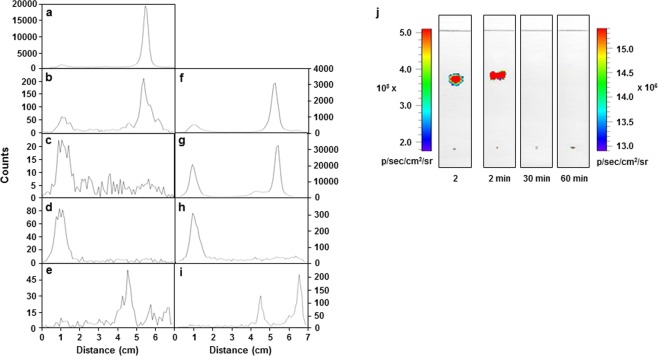
Radio-TLC analysis of the brain homogenates and blood samples obtained at 2, 30, and 60 min after injection of [^18^F]**2** into ICR mice. [^18^F]**2** (**a**); brain samples at 2 min (**b**), 30 min (**c**), and 60 min (**d**); blood samples at 2 min (**f**), 30 min (**g**), and 60 min (**h**). The 60-min samples (**d** and **h**) were further analyzed by radio-TLC using a 1:1:0.01 mixture of dichloromethane–methanol–triethylamine as the developing solvents (**e** and **i**). TLC analysis followed by fluorescence detection of the brain homogenates obtained at 2, 30, and 60 min after injection of **2** into Balb/C nude mice (**j**). The sample was spotted 1 cm from the bottom and the solvent front moved 7 cm from the bottom using a 4:1 mixture of ethyl acetate–hexane.

Radio-TLC data of the brain homogenates showed that most of [^18^F]**2** remained in the brain 2 min after injection (84%; Fig. 9a,b). However, the radioligand disappeared almost entirely, and the new polar radioactive peaks appeared at the origin of radio-TLC at 30 and 60 min after injection (82% and 100%, respectively; Fig. 9c,d). The radioligand also disappeared in the blood samples, but at a rate slower than that of the brain samples (Fig. 9f–h). The polar radioactive products were further analyzed by radio-TLC using a polar developing solvent system, and the polar products moved toward the solvent front. This showed that the polar products did not contain free [^18^F]fluoride ion, which would remain at the origin of the TLC, even in a polar developing solvent system (Fig. 9e,i)^13,14^. Moreover, the polar products were not adsorbed onto calcium phosphate (7% in calcium phosphate pellet vs. 93% in supernatant), which is a component of the bone matrix. This finding indicated that [^18^F]**2** did not undergo metabolic defluorination *in vivo*^13,14^. This result was consistent with low bone uptake in mice (Table 1).

In order to further investigate the metabolites of [^18^F]**2**, metabolism study of **2** was also performed; the brain homogenates were analyzed by TLC, followed by fluorescence detection. TLC data showed that a fluorescent spot was detected at the same position as that of **2** in the 2-min sample, which did not appear in the 30- and 60-min samples (Fig. 9j). This result was consistent with those of optical imaging, but not with radio-TLC data of [^18^F]**2** (Figs 8 and 9b–d).

## Discussion

Difluoroboron-curcumin derivatives, such as CRANAD-2, have been shown to label Aβ plaques in transgenic mouse brain and have potential for NIR imaging of Aβ plaques^6^. In order to extend this class of ligands to radioligands for PET imaging, we attempted a ^18^F/^19^F exchange reaction on one of the fluorine atoms of difluoroboron-curcumin. This is a well-established ^18^F/^19^F exchange reaction on BODIPY dye^15–17^. The isotope exchange reaction on the difluoroboron complex of 2,4-pentanedione, a center fragment of curcumin, only afforded ^18^F-labeled product when a methyl or ethyl group was substituted at C3, but with high instability (data not shown). Therefore, we synthesized four difluoroboron-curcumin derivatives for the development of ^18^F-labeled ligands (Fig. 1). These ligands (**1**–**4**) have substituents, such as 2-(2-fluoroethoxy)ethoxy or 3-fluoro-2-hydroxypropoxy group at the *para*-position of one of the phenyl rings (Figs 2–4). These substituents have been introduced to radioligands for improvement of *in vivo* properties as well as radiolabeling with ^18^F, as shown in the studies of radioligands for Aβ plaque imaging^18–20^.

For synthesis of [^18^F]**2**, a one-step [^18^F]fluorination was performed using the tosylate precursor of the difluoroboron-curcumin derivative. However, this synthesis had very low yield (0–16% based on radio-TLC). As an alternative, we synthesized a precursor, which was obtained from several steps including the reaction with (±)-epichlorohydrin. However, the epoxide ring opening by *n*-Bu_4_N[^18^F]F was not successful^20,21^. Therefore, a two-step reaction consisting of [^18^F]fluorination of **10**, followed by aldol condensation with **8** was conducted and gave [^18^F]**2** in relatively high yield (Fig. 5).

Based on the binding constants of the ligands for Aβ(1–42) aggregates, ligand **2** was selected for *in vitro* and *in vivo* studies. To examine whether **2** could label Aβ plaques *in vivo*, fluorescent staining of Aβ plaques was performed using the double transgenic mouse brain sections. The Aβ plaques in the cerebral cortex and hippocampus regions of the mouse brain sections were stained with ligand **2**, and the plaques in the same regions of the adjacent brain sections were also stained with an Aβ-specific antibody (Fig. 6a–f). This result indicated that ligand **2** distinctively stained Aβ plaques in transgenic mouse brain. In order to predict brain permeability of [^18^F]**2**, partition coefficient of the radioligand was measured. Optimal lipophilicity is one of important properties required for brain imaging ligand. Radioligands with measured log P values of 0.1–3.5 are known to penetrate the blood-brain barrier^22^. Therefore, [^18^F]**2** appeared to have favorable brain permeability (log P = 2.58 ± 0.04). The radioligand was further evaluated to predict its *in vivo* stability. Radio-TLC data showed that [^18^F]**2** was stable in both PBS and FBS (>83%) (Fig. 7). The result of [^18^F]**2** in FBS was consistent with the reported result of curcumin, in that curcumin was stable in a cell culture medium containing 10% fetal calf serum and in human blood (>80% at 1 h)^23^. However, the result of [^18^F]**2** in PBS is unlike curcumin, which was degraded to trans-6-(4′-hydroxy-3′-methoxyphenyl)-2,4-dioxo-5-hexenal, vanillin, ferulic acid, and feruloyl methane in phosphate buffer (pH 7.2)^23^.

The *in vitro* studies demonstrated that [^18^F]**2** satisfied the criteria required for development of Aβ imaging radioligand, and thus, the radioligand was evaluated *in vivo*. Brain imaging radioligand should have desirable brain pharmacokinetics in normal mice with high brain uptake at early time points after injection (>5% ID/g at 2 min) and fast wash-out from the brain by 30 min (<30% of initial uptake), as there are no Aβ plaques in the normal mouse brain^22,24^. However, the biodistribution study of [^18^F]**2** in normal mice showed poor brain uptake 2 min after injection, which increased over time (Table 1). These unusual brain pharmacokinetic data suggest that [^18^F]**2** may be converted to polar radioactive products in the mouse brain.

In order to investigate whether **2** exhibits the same brain pharmacokinetics as that of [^18^F]**2**, optical images of Balb/C nude mice were acquired. Optical images of the mouse brain showed moderate fluorescence signal intensity at 2 min after injection with the background signal intensity within 30 min (Fig. 8a,b). *Ex vivo* optical images displayed strong signal intensity in the liver and moderate signal intensities in the organs of elimination, such as small and large intestines at 2 min after injection. However, the fluorescence signals decreased significantly within 30 min (Fig. 8c). This result, therefore, suggests that ligand **2** may be rapidly converted to blue-shifted fluorescent products relative to emission of the ligand, and thus the excitation and emission wavelengths used for optical imaging may be no longer optimal for detection of the products formed from **2**.

The optical imaging data were not consistent with biodistribution data of mice injected with [^18^F]**2** (Fig. 8 and Table 1). Radioligand [^18^F]**2** and its non-radioactive equivalent (**2**) are the same compound with the difference of one neutron, and thus they would have the same metabolites *in vivo*. Therefore, metabolism study of [^18^F]**2** and **2** was performed, and the data were analyzed using radioactivity and fluorescence, respectively. Samples of the brain and blood of ICR mice were homogenized in acetonitrile to extract out the polar radioactive products, as well as non-polar products, and the supernatants were analyzed. The metabolism study performed using radioactivity showed that most of [^18^F]**2** remained in the brain and blood 2 min after injection, but it was converted to the polar radioactive products within 30 min after injection (Fig. 9). This result was consistent with the biodistribution data of [^18^F]**2** (Table 1). In contrast, the results obtained using fluorescence showed that **2** remained intact in the brain at 2 min after injection but disappeared within 30 min (Fig. 9j). This discrepancy between the optical images and biodistribution data is likely due to the formation of the metabolites from a hybrid PET/fluorescent ligand and their two different detection methods, such as radioactivity and fluorescence.

The results of the *in vivo* studies suggest that the polar products may have been formed from [^18^F]**2** in the mouse brain via two proposed pathways; either formation of polar ^18^F-labeled blue-shifted fluorescent products or formation of polar ^18^F-labeled metabolites derived from 2-(2-[^18^F]fluoroethoxy)ethoxy group with rapid wash-out of the rest non-radioactive product. In both cases, the radioactivity in the brain would increase, but the fluorescence signal would disappear over time. Although the polar radioactive products were not identified, it is unlikely that 2-[^18^F]fluoroethanol and 2-(2-[^18^F]fluoroethoxy)ethanol are the metabolites of [^18^F]**2**, in that 2-fluoroethanol did not match with any of the polar radioactive products on TLC and it is more polar than 2-(2-fluoroethoxy)ethanol (Supplementary Table S1). Moreover, the polar radioactive products did not contain the proposed degradation products, such as 4-(2-(2-fluoroethoxy)ethoxy) benzoic acid and 4-(2-(2-fluoroethoxy)ethoxy)cinnamic acid (Supplementary Table S1). These characteristics are unlike those of similar degradation products that were shown in curcumin^23^. In addition, there are quite a few radioligands substituted with 2-(2-[^18^F]fluoroethoxy)ethoxy or 2-(2-(2[^18^F]fluoroethoxy)ethoxy)ethoxy group^22,25–27^. These polyethylene glycol groups are known to control lipophilicity and improve bioavailability of radioligands, and thus they have been introduced to the radioligands for Aβ imaging^22,26,27^. This result led us to propose that [^18^F]**2** may be converted to polar ^18^F-labeled blue-shifted fluorescent products.

The *in vivo* characteristics of [^18^F]**2** are distinct from those of ^18^F-labeled curcumin derivatives. In our previous studies, 4′-*O*-[^18^F]fluoropropylcurcumin had poor brain permeability at 2 min after injection in normal mice, but showed rapid wash-out from the brain within 30 min. Moreover, the radioligand was intact in the mouse brain once it was taken up by the brain^3^. Similarly, ligand **2** may have different *in vivo* properties from CRANAD-2, which has dimethylamino groups at both phenyl rings^6^. Therefore, further studies are warranted to identify the polar products formed from [^18^F]**2** *in vivo*.

## Conclusion

Of the four difluoroboron-curcumin derivatives, ligand **2** was selected for radiolabeling, *in vitro* and *in vivo* evaluation. Although the ligand was able to distinctively stain Aβ plaques in transgenic mouse brain sections and had suitable lipophilicity, the *in vivo* studies of [^18^F]**2** did not show favorable brain pharmacokinetics in normal mice. Although the polar radioactive products formed from [^18^F]**2** need to be identified, the results of this study would serve as a starting point for the design of metabolically stable ^18^F-labeled difluoroboron-curcumin derivatives for Aβ imaging.

## Materials and Methods

### Materials and equipment for synthesis of non-radioactive ligands

Reagents including CRANAD-2 were purchased from Merck (Darmstadt, Germany) and 4-(methylamino)benzaldehyde was from Manchester Organics (Cheshire, UK). β-Amyloid(1–42) peptide was purchased from Bachem (Torrance, CA, USA). ^1^H NMR spectra were obtained using a Bruker Avance III 500 (500 MHz) spectrometer (Rheinstetten, Germany), and chemical shifts (δ) were reported as the ppm downfield of the internal tetramethylsilane. ^19^F NMR spectra were obtained using a Bruker Avance III HD (300 MHz) spectrometer. The electron impact (EI) and fast atom bombardment (FAB) mass spectra were obtained using a JMS-700 Mstation (JEOL Ltd, Tokyo, Japan). Purification of the non-radioactive ligands was performed using HPLC (Thermo Scientific, Waltham, MA, USA) that was equipped with a semi-preparative column (YMC-Pack C18, 10 × 250 mm, 5 µm). Purity of the ligands was determined using HPLC equipped with an analytical column (YMC-Pack C18, 4.6 × 250 mm, 5 µm) (Supplementary Figs S9–S12). The eluent was monitored using a UV (254 nm) detector.

### (1E,4Z)-1-(4-(Methylamino)phenyl)- and (1E,4Z)-1-(4-(dimethylamino)phenyl)-5-hydroxyhexa-1,4-dien-3-one (5 and 6)

B_2_O_3_ (2.33 g, 33.5 mmol) was added to a solution of 2,4-pentanedione (3.03 mL, 29.5 mmol) in ethyl acetate. The solution was then stirred at 80 °C for 30 min. Next, 4-(methylamino)benzaldehyde (or 4-(dimethylamino)benzaldehyde) (13.4 mmol) in ethyl acetate (2.7 mL) was added to the solution. After stirring at 80 °C for 30 min, *n*-butylamine (0.66 mL, 6.70 mmol) was added dropwise to the mixture. The reaction mixture was allowed to stir at 100 °C for 1 h, cooled to rt, and then treated with 0.4 N HCl (10 mL) at 50 °C for 30 min. After the reaction was quenched with saturated NaHCO_3_ solution, the reaction mixture was extracted with ethyl acetate, washed with water, and then dried over Na_2_SO_4_. Flash column chromatography (4:1 hexane-ethyl acetate) gave **5** (500 mg, 17.2%) or **6** (560.8 mg, 18.1%) as a yellow solid.

**5:** ^1^H NMR (CDCl_3_) *δ* 15.65 (s, 1H), 7.55 (d, *J* = 16 Hz, 1H), 7.39 (d, *J* = 8.5 Hz, 2H), 6.58 (d, *J* = 8.5 Hz, 2H), 6.28 (d, *J* = 15.5 Hz, 1H), 5.59 (s, 1H), 2.88 (s, 3H), 2.13 (s, 3H); MS (EI) *m*/*z* 217 (M^+^): HRMS calcd for C_13_H_15_NO_2_, 217.1103; found, 217.1105.

**6:** ^1^H NMR (CDCl_3_) *δ* 7.57 (d, *J* = 16 Hz, 1H), 7.44 (d, *J* = 9 Hz, 2H), 6.71 (br d, *J* = 8.5 Hz, 2 H), 6.29 (d, *J* = 16 Hz, 1H), 5.59 (s, 1H), 3.04 (s, 6H), 2.13 (s, 3 H); MS (EI) *m*/*z* 231 (M^+^): HRMS calcd for C_14_H_17_NO_2_, 231.1259; found, 231.1260.

### BF_2_ complexes of 5 and 6 (7 and 8)

Compound **5** (or **6**) (0.86 mmol) was dissolved in 10 mL dichloromethane, and BF_3_·Et_2_O (0.67 mL, 5.19 mmol) was added to this solution in a dropwise fashion. The reaction mixture was stirred at rt for 3 h. After the reaction was quenched with saturated NaHCO_3_ solution, the reaction mixture was extracted with ethyl acetate, washed with water, and then dried over Na_2_SO_4_. Flash column chromatography (2:1 hexane-ethyl acetate) gave **7** (200 mg, 87.7%) or **8** (230 mg, 95.3%) as a red solid.

**7:** ^1^H NMR (CDCl_3_) δ 8.04 (d, *J* = 15.5 Hz, 1H), 7.48 (d. *J* = 9 Hz, 2H), 6.59 (d, *J* = 8.5 Hz, 2H), 6.39 (d, *J* = 15 Hz, 1H), 5.87 (s. 1H), 4.43 (s br, 1H), 2.93 (s. 3H), 2.27 (s, 3H); MS (EI) *m*/*z* 265 (M^+^): HRMS calcd for C_13_H_14_BF_2_NO_2_, 265.1086; found, 265.1088.

**8:** ^1^H NMR (CDCl_3_) δ 8.06 (d, *J* = 15.5 Hz, 1H), 7.52 (d, *J* = 9 Hz, 2H), 6.69 (d, *J* = 9 Hz, 2H), 6.39 (d, *J* = 15 Hz, 1H), 5.87 (s, 1H), 3.09 (s, 6H), 2.26 (s, 3H); MS (EI) *m*/*z* 279 (M^+^): HRMS calcd for C_14_H_16_BF_2_NO_2_, 279.1242; found, 279.1239.

### 4-(2-(2-Hydroxyethoxy)ethoxy)benzaldehyde (9)

4-Hydroxybenzaldehyde (500 mg, 4.09 mmol) and K_2_CO_3_ (848 mg, 6.14 mmol) were dissolved in 20 mL DMF, and the solution was stirred at rt for 15 min. After addition of 2-(2-chloroethoxy)ethanol (0.86 mL, 8.19 mmol), the reaction mixture was stirred at 100 °C overnight. The reaction mixture was extracted with ethyl acetate and water, and the organic layer was washed with water and then with saturated NH_4_Cl solution, and dried over Na_2_SO_4_. Flash column chromatography (1:1 hexane-ethyl acetate) gave **9** (600 mg, 69.8%) as a colorless oil. ^1^H NMR (CDCl_3_) δ 9.89 (s, 1H), 7.85 (d, *J* = 9 Hz, 2H), 7.04 (d, *J* = 8.5 Hz, 2H), 4.24 (t, *J* = 4.5 Hz, 2H), 3.91 (t, *J* = 3.5 Hz, 2H), 3.79 (t, *J* = 5 Hz, 2H), 3.70 (t, *J* = 3.5 Hz, 2H); MS (EI) *m*/*z* 210 (M^+^): HRMS calcd for C_11_H_14_O_4_, 210.0892; found, 210.0895.

### 2-(2-(4-Formylphenoxy)ethoxy)ethyl toluenesulfonate (10)

Compound **9** (332 mg, 1.58 mmol) was dissolved in 2 mL dichloromethane, and then *p*-toluenesulfonyl chloride (361.5 mg, 1.90 mmol) was added to this solution. After the addition of triethylamine (1.32 mL, 9.49 mmol) at 0 °C, the reaction mixture was stirred at rt overnight. The reaction was then quenched with saturated NH_4_Cl solution, and the reaction mixture was extracted with dichloromethane, washed with water, and then dried over Na_2_SO_4_. Flash column chromatography (1:1 hexane-ethyl acetate) gave **10** (480 mg, 83.5%) as a white solid. ^1^H NMR (CDCl_3_) δ 9.89 (s, 1H), 7.84 (d, *J* = 9 Hz, 2H), 7.80 (d, *J* = 8.5 Hz, 2H), 7.31 (d, *J* = 8 Hz, 2H), 7.00 (d, *J* = 8.5 Hz, 2H), 4.20 (t, *J* = 3.5 Hz, 2H), 4.15 (t, *J* = 5 Hz, 2H), 3.84 (t, *J* = 3.5 Hz, 2H), 3.78 (t, *J* = 3.5 Hz, 2H), 2.41 (s, 3H); MS (EI) *m*/*z* 364 (M^+^): HRMS calcd for C_18_H_20_O_6_S, 364.0981; found, 364.0984.

### 4-(2-(2-Fluoroethoxy)ethoxy)benzaldehyde (11)

Compound **10** (427 mg, 1.17 mmol) was dissolved in 20 mL *t*-BuOH, and CsF (670 mg, 4.41 mmol) was added to this solution. The reaction mixture was stirred at 100 °C overnight. The mixture was then extracted with ethyl acetate and water, and the organic layer was washed with water and dried over Na_2_SO_4_. Flash column chromatography (1:2 hexane-ethyl acetate) gave **11** (170 mg, 68.4%) as a colorless oil. ^1^H NMR (CDCl_3_) δ 9.89 (s.1H), 7.84 (d, *J* = 8.5 Hz, 2H), 7.04 (d, *J* = 9 Hz, 2H), 4.65 (dt, *J* = 47.5 and 3 Hz, 2H), 4.24 (t, *J* = 4.5 Hz, 2H), 3.94 (t, *J* = 5 Hz, 2H), 3.87 (dt, *J* = 29.5 and 3 Hz, 2H); MS (EI) *m*/*z* 212 (M^+^): HRMS calcd for C_11_H_13_FO_3_, 212.0849; found, 212.0848.

### BF_2_ complexes of 4′-monomethylamino- and 4′-dimethylamino-4″-(2-(2-fluoroethoxy)ethoxy)curcuminoid (1 and 2)

Compound **11** (20 mg, 0.09 mmol) was added to **7** (or **8**) (0.13 mmol) dissolved in 1.5 mL ethyl acetate. After the reaction solution was stirred at rt for 10 min, piperidine (17.8 μL, 0.18 mmol) was added dropwise. The reaction mixture was then allowed to stir at 110 °C for 20 min. The mixture was then extracted with ethyl acetate and water, and the organic layer was washed with water and dried over Na_2_SO_4_. Flash column chromatography (2:1 hexane-ethyl acetate) gave **1** (10 mg, 16.9%) or **2** (20 mg, 32.3%) as a violet solid. Ligands **1** and 2 were further purified by HPLC using a semi-preparative column eluted with a 30:70 mixture of 0.1% trifluoroacetic acid (TFA, aq.) and acetonitrile at a flow rate of 4 mL/min.

**1:** ^1^H NMR (CDCl_3_) δ 8.03 (d, *J* = 15 Hz, 1H), 7.96 (d, *J* = 15.5 Hz, 1H), 7.56 (d, *J* = 9 Hz, 2H), 7.50 (d, *J* = 8.5 Hz, 2H), 6.96 (d, *J* = 8.5 Hz, 2H), 6.60 (d, *J* = 8.5 Hz, 2H), 6.57 (d, *J* = 15.5 Hz, 1H), 6.48 (d, *J* = 15.5 Hz, 1H), 5.94 (s, 1H), 4.65 (dt, *J* = 47.5 and 4 Hz, 2H), 4.21 (t, *J* = 5 Hz, 2H), 3.93 (t, *J* = 4.5 Hz, 2H), 3.86 (dt, *J* = 29.5 and 4.5 Hz, 2H), 2.93 (s, 3H); ^19^F NMR (CDCl_3_) δ −141.69, −141.75, −222.91; MS (FAB) *m*/*z* 459 (M^+^): HRMS calcd for C_24_H_25_BF_3_NO_4_, 459.1829; found, 459.1832.

**2:** ^1^H NMR (CDCl_3_) δ 8.04 (d, *J* = 15 Hz, 1H), 7.95 (d, *J* = 15.5 Hz, 1H), 7.56 (q, *J* = 9 Hz, 4H), 6.96 (d, *J* = 9 Hz, 2H), 6.79 (d, *J* = 9 Hz, 2H), 6.57 (d, *J* = 15.5 Hz, 1H), 6.50 (d, *J* = 15.5 Hz, 1H), 5.94 (s, 1H), 4.66 (dt, *J* = 48 and 3.5 Hz, 2H), 4.21 (t, *J* = 4.5 Hz, 2H), 3.93 (t, *J* = 5 Hz, 2H), 3.86 (dt, *J* = 29.5 and 4 Hz, 2H), 3.10 (s, 6H); ^19^F NMR (CDCl_3_) δ −141.71, −141.77, −222.90; MS (FAB) *m*/*z* 473 (M^+^): HRMS calcd for C_25_H_27_BF_3_NO_4_, 473.1985; found, 473.1985.

### 4-(2-(2-Hydroxyethoxy)ethoxy)-3-methoxybenzaldehyde (12)

Vanillin (1 g, 6.57 mmol) and K_2_CO_3_ (1.36 g, 9.85 mmol) were dissolved in 10 mL DMF, and the solution was stirred at rt for 15 min. After addition of 2-(2-chloroethoxy)ethanol (1.38 mL, 13.14 mmol), the reaction mixture was stirred at 100 °C overnight. The reaction mixture was extracted with ethyl acetate and water, and the organic layer was washed with water and then with saturated NH_4_Cl solution, and dried over Na_2_SO_4_. Flash column chromatography (1:1 hexane-ethyl acetate) gave **12** (1.3 g, 82.3%) as a colorless oil. ^1^H NMR (CDCl_3_) δ 9.85 (s, 1H), 7.45 (dd, *J* = 10 and 2 Hz, 1H), 7.42 (d, *J* = 2 Hz, 1H), 7.00 (d, *J* = 8 Hz, 1H), 4.27 (t, *J* = 4.5 Hz, 2H), 3.97 (*t*, *J* = 4.5 Hz, 2H), 3.93 (s, 3H), 3.78 (t, *J* = 4 Hz, 2H), 3.71 (t, *J* = 4 Hz, 2H); MS (EI) *m*/*z* 240 (M^+^): HRMS calcd for C_12_H_16_O_5_, 240.0998; found, 240.0999.

### 2-(2-(4-Formyl-2-methoxyphenoxy)ethoxy)ethyl toluenesulfonate (13)

Compound **12** (500 mg, 2.08 mmol) was dissolved in 7 mL dichloromethane, and then *p*-toluenesulfonyl chloride (476 mg, 2.49 mmol) was added to this solution. After the addition of triethylamine (1.74 mL, 12.48 mmol) at 0 °C, the reaction mixture was stirred at rt overnight. The reaction was then quenched with saturated NH_4_Cl solution, and the reaction mixture was extracted with dichloromethane, washed with water, and then dried over Na_2_SO_4_. Flash column chromatography (1:1 hexane-ethyl acetate) gave **13** (700 mg, 85.3%) as a white solid. ^1^H NMR (CDCl_3_) δ 9.85 (s, 1H), 7.80 (d, *J* = 12 Hz, 2H), 7.44 (dd, *J* = 10 and 1.5 Hz, 1H), 7.41 (d, *J* = 2 Hz, 1H), 7.31 (d, *J* = 8 Hz, 2H), 6.99 (d, *J* = 8.5 Hz, 1H), 4.2 (br q, *J* = 4 Hz, 4H), 3.90 (s, 3H), 3.87 (t, *J* = 4 Hz, 2H), 3.78 (t, *J* = 5 Hz, 2H), 2.42 (s, 3H); MS (EI) *m*/*z* 394 (M^+^): HRMS calcd for C_19_H_22_O_7_S, 394.1086; found, 394.1083.

### 4-(2-(2-Fluoroethoxy)ethoxy)-3-methoxybenzaldehyde (14)

Compound **13** (200 mg, 0.50 mmol) was dissolved in 10 mL *t*-BuOH, and CsF (231 mg, 1.52 mmol) was added to this solution. The reaction mixture was stirred at 100 °C overnight. At the end of reaction, the mixture was extracted with ethyl acetate and water, and the organic layer was washed with water and then dried over Na_2_SO_4_. Flash column chromatography (1:2 hexane-ethyl acetate) gave **14** (70 mg, 56.9%) as a colorless oil. ^1^H NMR (CDCl_3_) δ 9.86 (s, 1H), 7.45 (dd, *J* = 8 and 1.5 Hz, 1H), 7.42 (d, *J* = 2 Hz, 1H), 7.03 (d, *J* = 8 Hz, 1H), 4.65 (dt, *J* = 47.5 and 4 Hz, 2H), 4.3 (t, 4.5 Hz, 2H), 3.98 (t, 5 Hz, 2H), 3.93 (s, 3H), 3.87 (dt, *J* = 26.5 and 5 Hz, 2H); MS (EI) *m*/*z* 242 (M^+^): HRMS calcd for C_12_H_15_FO_4_, 242.0954; found, 242.0951.

### (1E,4Z)-5-Hydroxy-1-(4-hydroxy-3-methoxyphenyl)hexa-1,4-dien-3-one (15)

B_2_O_3_ (0.8 g, 11.5 mmol) was added to a solution of 2,4-pentanedione (1.48 mL, 14.4 mmol) in ethyl acetate. This solution was then stirred at 80 °C for 30 min. Vanillin (1 g, 6.57 mmol) and (*n*-BuO)_3_B (0.5 mL, 1.85 mmol) in ethyl acetate (4 mL) were added to this reaction solution. After stirring at 80 °C for 30 min, *n*-butylamine (0.66 mL, 6.70 mmol) was added dropwise to the mixture, which was then allowed to stir at 100 °C for 1 h. The reaction mixture was then treated with 0.4 N HCl (10 mL) at 50 °C for 30 min. After the reaction was then quenched with saturated NaHCO_3_ solution, the reaction mixture was extracted with ethyl acetate, washed with water, and then dried over Na_2_SO_4_. Flash column chromatography (4:1 hexane-ethyl acetate) gave **15** (300 mg, 19.5%) as a yellow solid. ^1^H NMR (CDCl_3_) δ 7.54 (d, *J* = 15.5 Hz, 1H), 7.10 (dd, *J* = 8 and 2 Hz, 1H), 7.02 (d, *J* = 2 Hz, 1H), 6.93 (d, *J* = 8.5 Hz, 1H), 6.34 (d, *J* = 16 Hz, 1H), 5.83 (s, 1H), 3.94 (s, 3H), 2.15 (s, 3H); MS (EI) *m*/*z* 234 (M^+^): HRMS calcd for C_13_H_14_O_4_, 234.0892; found, 234.0893.

### BF_2_ complex of 15 (16)

Compound **15** (0.2 g, 0.85 mmol) was dissolved in 15 mL dichloromethane, and BF_3_·Et_2_O (0.83 mL, 5.12 mmol) was added to this solution dropwise. The reaction mixture was stirred at rt for 2 h. After the reaction was quenched with saturated NaHCO_3_ solution, the reaction mixture was extracted with ethyl acetate, washed with water, and then dried over Na_2_SO_4_. Flash column chromatography (2:1 hexane-ethyl acetate) gave **16** (150 mg, 62.2%) as a red solid. ^1^H NMR (CDCl_3_) δ 8.03 (d, *J* = 15.5 Hz, 1H), 7.24 (dd, *J* = 8.5 and 2 Hz, 1H), 7.08 (d, *J* = 1.5 Hz, 1H), 6.98 (d, *J* = 8 Hz, 1H), 6.51 (d, *J* = 15.5, 1H), 6.04 (s, 1H), 5.97 (s, 1H), 3.96 (s, 3H), 2.32 (s, 3H); MS (EI) *m*/*z* 282 (M^+^): HRMS calcd for C_13_H_13_BF_2_O_4_ 282.0877; found, 282.0875.

### BF_2_ complex of 4′-(2-(2-fluoroethoxy)ethoxy)curcuminoid (3)

Compound **16** (30 mg, 0.10 mmol) was dissolved in 1.5 mL ethyl acetate, and then (*n*-BuO)_3_B (59.2 μL,0.2 mmol) and compound **14** (21.4 mg, 0.08 mmol) were sequentially added to this solution. After stirring at rt for 10 min, piperidine (5.2 μL, 0.05 mmol) was added dropwise to the mixture. The reaction mixture was then allowed to stir at 110 °C for 20 min. At the end of reaction, the mixture was extracted with ethyl acetate and water, and the organic layer was washed with water and then dried over Na_2_SO_4_. Flash column chromatography (1:1 hexane-ethyl acetate) gave **3** (18.0 mg, 33.5%) as a red solid. Ligand **3** was further purified by HPLC using a semi-preparative column eluted with a 40:60 mixture of 0.1% TFA (aq.) and acetonitrile at a flow rate of 3 mL/min. ^1^H NMR (CDCl_3_) δ 7.98 (d, *J* = 15 Hz, 2H), 7.22 (t, *J* = 8.5 Hz, 2H), 7.09 (d, *J* = 9 Hz, 2H), 6.97 (q, *J* = 8 Hz, 2H), 6.58 (dd, *J* = 15.5 and 5.5 Hz, 2H), 6.02 (s, 1H), 4.65 (dt, *J* = 47.5 and 4 Hz, 2H), 4.27 (t, *J* = 4.5 Hz, 2H), 3.96 (m, 5H), 3.91 (s, 3H), 3.87 (dt, *J* = 30 Hz, 4.5 Hz, 2H); ^19^F NMR (CDCl_3_) δ −141.04, −141.10, −222.99; MS (FAB) *m*/*z* 506 (M^+^): HRMS calcd for C_25_H_26_BF_3_O_7_, 506.1724; found, 506.1728.

### 4-(2,3-Dihydroxypropoxy)benzaldehyde (17)

4-Hydroxybenzaldehyde (500 mg, 4.09 mmol) was dissolved in 40 mL EtOH, and NaOH (246 mg, 6.14 mmol) in 4 mL water was added to this solution. After the solution was stirred at rt for 10 min, (±)-3-chloro-1,2-propanediol (0.479 mL, 5.73 mmol) was added dropwise. The reaction mixture was stirred at 100 °C overnight. At the end of reaction, the reaction mixture was filtered and the filtrate was extracted with ethyl acetate, washed with water, and then dried over Na_2_SO_4_. Flash column chromatography (1:2 hexane-ethyl acetate) gave **17** (500 mg, 62.3%) as a white solid. ^1^H NMR (CDCl_3_) δ 9.90 (s, 1H), 7.86 (d, *J* = 9 Hz, 2H), 7.05 (d, *J* = 8.5 Hz, 2H), 4.13–4.17 (m, 3H), 3.87–3.89 (m, 1H), 3.76–3.80 (m, 1H); MS (EI) *m*/*z* 196 (M^+^): HRMS calcd for C_10_H_12_O_4_, 196.0736; found, 196.0737.

### 3-(4-Formylphenoxy)-2-hydroxypropyl toluenesulfonate (18)

Compound **17** (450 mg, 2.30 mmol) was dissolved in 30 mL dichloromethane, and triethylamine (1.92 mL, 13.77 mmol) was added to this solution at 0 °C. After stirring at rt for 10 min, *p*-toluenesulfonyl chloride (525 mg, 2.75 mmol) was added to the reaction mixture, which was then allowed to stir at rt overnight. The reaction mixture was extracted with dichloromethane and water, and the organic layer was washed with water and then dried over Na_2_SO_4_. Flash column chromatography (1:1 hexane-ethyl acetate) gave **18** (286 mg, 35.5%) as a white solid. ^1^H NMR (CDCl_3_) δ 9.90 (s, 1H), 7.85 (d, *J* = 9 Hz, 2H), 7.81 (d, *J* = 8.5 Hz, 2H), 7.33 (d, *J* = 8 Hz, 2H), 6.96 (d, *J* = 8 Hz, 2H), 4.19–4.28 (m, 3H), 4.09 (d, *J* = 5 Hz, 2H), 2.43 (s, 3H); MS (EI) *m*/*z* 350 (M^+^): HRMS calcd for C_17_H_18_O_6_S, 350.0824; found, 350.0825.

### 3-(4-Formylphenoxy)-2-((tetrahydro-2H-pyran-2-yl)oxy)propyl toluenesulfonate (19)

Compound **18** (250 mg, 0.71 mmol) was dissolved in 15 mL dichloromethane. 3,4-Dihydro-2*H*-pyran (0.78 mL, 8.57 mmol) and pyridinium *p*-toluenesulfonate (1.08 g, 4.28 mmol) were added to this solution sequentially. The reaction mixture was refluxed for 4 h. At the end of reaction, the mixture was extracted with ethyl acetate and water, and the organic layer was washed with water and then dried over Na_2_SO_4_. Flash column chromatography (1:1 hexane-ethyl acetate) gave **19** (262 mg, 84.5%) as a colorless oil. ^1^H NMR (CDCl_3_) δ 9.90 (s, 1H), 7.83 (d, *J* = 8.5 Hz, 2H), 7.77 (d, *J* = 8.5 Hz, 2H), 7.29 (d, *J* = 8 Hz, 2H), 6.90–6.93 (m, 2H), 4.69–4.71 (m, 1H), 4.29–4.32 (m, 1H), 4.16–4.26 (m, 3H), 4.06–4.12 (m, 1H), 3.78–3.89 (m, 1H), 3.47–3.50 (m, 1H), 2.41 (s, 3H), 1.67–1.77 (m, 2 H), 1.48–1.57 (m, 4H); MS (EI) *m*/*z* 434 (M^+^): HRMS calcd for C_22_H_26_O_7_S, 434.1399; found, 434.1399.

### 4-(3-Fluoro-2-hydroxypropoxy)benzaldehyde (20)

Compound **19** (60 mg, 0.14 mmol) was dissolved in 2 mL *t*-BuOH, and CsF (50.2 mg, 0.33 mmol) was added to this solution. After the reaction mixture was stirred at 110 °C overnight, it was extracted with ethyl acetate, washed with water, dried over Na_2_SO_4_, and then concentrated *in vacuo*.

The residue was dissolved in 1.5 mL acetonitrile, and 0.2 mL 1 N HCl was added to the solution. The reaction mixture was stirred at 100 °C for 5 min. After the reaction was quenched with 0.4 mL saturated NaHCO_3_ solution, the reaction mixture was extracted with ethyl acetate, washed with water, and then dried over Na_2_SO_4_. Flash column chromatography (1:1 hexane-ethyl acetate) gave **20** (20 mg, 72.1%) as a colorless oil. ^1^H NMR (CDCl_3_) δ 9.91 (s, 1H), 7.87 (d, *J* = 9 Hz, 2H), 7.05 (d, *J* = 9 Hz, 2H), 4.69 (ds, *J* = 47 Hz and 4.5 Hz, 2H), 4.27–4.33 (m, 1H), 4.14–4.20 (m, 2H), 2.38 (s, 1H); MS (EI) *m*/*z* 198 (M^+^): HRMS calcd for C_10_H_11_FO_3_, 198.0692; found, 198.0693.

### BF_2_ complex of 4′-dimethylamino-4″-(3-fluoro-2-hydroxypropoxy)curcuminoid (4)

Compound **8** (23.6 mg, 0.08 mmol) was dissolved in 1.5 mL ethyl acetate, and compound **20** (14 mg, 0.08 mmol) was added to this solution. After stirring at rt for 10 min, piperidine (14 μL, 0.14 mmol) was added dropwise to the mixture, which was then allowed to stir at 110 °C for 20 min. The reaction mixture was extracted with ethyl acetate and water, and the organic layer was washed with water and then dried over Na_2_SO_4_. Flash column chromatography (1:1 hexane-ethyl acetate) gave **4** (8 mg, 21.8%) as a violet solid. Ligand **4** was further purified by HPLC using a semi-preparative column eluted with a 35:65 mixture of 0.1% TFA (aq.) and acetonitrile at a flow rate of 3 mL/min. ^1^H NMR (CDCl_3_) δ 8.06 (d, *J* = 15.5 Hz, 1H), 7.95 (d, *J* = 15.5 Hz, 1H), 7.57 (d, *J* = 9 Hz, 2H), 7.55 (d, *J* = 8.5 Hz, 2H), 6.97 (d, *J* = 8.5 Hz, 2H), 6.73–6.74 (m, 2H), 6.59 (d, *J* = 15.5 Hz, 1H), 6.50 (d, *J* = 15.5 Hz, 1H), 5.95 (s, 1H), 4.70 (ds, *J* = 47.5 Hz and 4.5 Hz, 2H), 4.25–4.32 (m, 1H), 4.11–4.16 (m, 2H), 3.12 (s, 6H); ^19^F NMR (CDCl_3_) δ −141.73, −141.79, −233.08; MS (FAB) *m*/*z* 459 (M^+^): HRMS calcd for C_24_H_25_BF_3_NO_4_, 459.1829; found, 459.1827.

### Materials and equipment for synthesis of radioligand

[^18^F]Fluoride was produced by the ^18^O(p,n)^18^F reaction using a GE Healthcare PETtrace cyclotron (Uppsala, Sweden). Radioactivity was measured in a dose calibrator (Biodex Medical Systems, Shirley, NY, USA). TLC was performed on Merck F_254_ silica plates and analyzed on a Bioscan radio-TLC scanner (Washington, D.C., USA). Purification and analysis of the radioligand were performed using HPLC equipped with a semi-preparative column (YMC-Pack C18, 10 × 250 mm, 5 µm) or an analytical column (YMC-Pack C18, 4.6 × 250 mm, 5 µm). The eluent was monitored simultaneously, using UV (254 nm) and NaI(T1) radioactivity detectors.

### Radioligand [^18^F]2

[^18^F]Fluoride (111–1110 MBq) was placed in a Vacutainer containing *n*-Bu_4_NHCO_3_. Three azeotropic distillations were then performed using 200–300 μL aliquots of acetonitrile at 90 °C (oil bath) under a gentle stream of N_2_. The resulting *n*-Bu_4_N[^18^F]F was then dissolved in acetonitrile (100 μL) and transferred to a reaction vial containing **10** (0.5 mg, 1.4 μmol). This reaction solution was allowed to stir at 110 °C for 10 min. The resulting mixture was extracted with ethyl acetate and water, and the organic layer was dried under a gentle stream of N_2_. The residue was re-dissolved in ethyl acetate (200 μL), which was then transferred to a reaction vial containing **8**. The reaction mixture was heated at 110 °C for 20 min after addition of *n*-butylamine (13.6 μL, 1.4 μmol). The mixture was cooled, diluted with water (2 mL), and extracted with ethyl acetate (2 mL). The organic layer was passed through a 3-cm Na_2_SO_4_ plug, and the solvent was removed under a stream of N_2_ at 50 °C (water bath). The reaction mixture was then purified by HPLC using a semi-preparative column eluted with a 35:65 mixture of 0.1% TFA (aq.) and acetonitrile at a flow rate of 3 mL/min. The desired product eluted between 23 and 24 min.

Molar activity was determined by comparing the UV peak area of the desired radioactive peak and the UV peak areas of different concentrations of non-radioactive ligand **2** on HPLC. This was performed using an analytical column eluted with a 25:75 mixture of 0.1% TFA (aq.) and acetonitrile at a flow rate of 1 mL/min. The radioligand [^18^F]**2** was identified by co-injecting the radioligand with non-radioactive ligand **2** into the HPLC system (Supplementary Fig. S14).

### Excitation and emission spectra

Ligand **2** was dissolved in methanol (2.5 μM), and its excitation and emission spectra were obtained using a Mithras^2^ LB 943 monochromator multimode microplate reader (Berthold Technologies, Bad Wildbad, Germany).

### *In vitro* binding assays using Aβ(1–42) aggregates

Aβ(1–42) aggregates were prepared as described in the literature^25^. Briefly, Aβ(1–42) peptide (200 μM) was dissolved in PBS and stirred gently at 37 °C for 2 d. The Aβ(1–42) aggregate formation was confirmed by thioflavin-T fluorescence assay. *In vitro* binding assays were carried out using the methods described in the literature with some modifications^6^. Various concentrations of ligands (10–400 nM) were dissolved in DMSO, which were then added to PBS solutions of Aβ(1–42) aggregates (1 μM) charged in 96-well black clear-bottom plates. The final concentration of DMSO in a total volume of solution was 1%. Non-specific binding was determined in the absence of Aβ(1–42) aggregates. The reaction mixtures were incubated at 37 °C for 1 h, after which the fluorescence spectra of the ligands were obtained (excitation 510–560 nm and emission 590–660 nm) using a monochromator multimode microplate reader. All experiments were performed in triplicate. K_d_ values were determined using GraphPad Prism software 5.

### Staining of Aβ plaques in double transgenic mouse brain sections

A double transgenic mouse (APP/PS1, male, 18 months old) was transcardially perfused with PBS, followed by 10% neutral buffered formalin (NBF) under anesthesia. The brain was then immediately removed from skull and post-fixed in 10% NBF for 24 h. The hippocampus and cerebral cortex regions were sliced using brain matrix, embedded in paraffin, and sectioned serially into 4 μm thickness. Brain sections from an age-matched wild-type mouse (male) were obtained as described above.

Slide-mounted brain sections were deparaffinized in xylene, rehydrated in graded ethanol, and transferred to PBS. The brain sections were incubated with ligand **2** (100 μM) in 50% ethanol/PBS for 10 min, washed with 50% ethanol, water and PBS, and coverslipped with a drop of ProLong Gold anti-fade reagent counterstained with DAPI (Invitrogen, Carlsbad, CA, USA).

For immunohistochemical localization of the Aβ plaques, the adjacent sections were deparaffinized as described above. Heat-induced antigen epitope retrieval was performed on the sections using citrate buffer (pH 6.0; Dako, Carpinteria, CA, USA) at 121 °C for 3 min to reveal hidden antigen epitopes. Endogenous peroxidase activity was then blocked with 3% hydrogen peroxide at rt for 10 min. After washing in PBS, the sections were treated with serum-free blocking solution (Dako) at rt for 20 min. Subsequently, the sections were incubated with mouse monoclonal anti-Aβ antibody (diluted 1:400; BioLegend, San Diego, CA, USA) at rt for 60 min. After washing in PBS, the sections were stained with Alexa Fluor 488-conjugated goat anti-mouse IgG (diluted 1:250, Invitrogen) at rt for 30 min. The sections were finally washed with PBS and coverslipped with a drop of ProLong Gold anti-fade reagent counterstained with DAPI.

The sections were examined using a confocal laser scanning microscope M780 (Carl Zeiss, Oberkochen, Germany) with a 10x or 20x plan-apochromat objective lens (NA = 0.45 or 0.8). The images were acquired using a 488-nm laser with a 491–588-nm filter for the immunostaining, and a 561-nm laser with a 589–735-nm filter for ligand **2**.

### Partition coefficient measurement

Partition coefficient of [^18^F]**2** was measured according to the method described in the literature^28^. [^18^F]**2** was dissolved in 1-octanol (1 mL) and diluted with water (1 mL). The solution was vortexed vigorously for 5 min and then centrifuged. The 1-octanol and aqueous layers were separated out, and a 500-μL aliquot of each layer was removed and measured using a gamma counter. Samples from the 1-octanol and aqueous layers were repartitioned until consistent values were obtained. The log P is expressed as the logarithm of the ratio of the counts per minute of 1-octanol versus that of water.

### *In vitro* stability study

[^18^F]**2** (21.5 MBq) was dissolved in a minimal volume of 10% ethanol–saline and incubated with PBS or 50% FBS (Gibco, Brooklyn, NY, USA) at 37 °C for 120 min. Aliquots were taken from the incubation solution after 0, 2, 30, 60, and 120 min. The aliquots were analyzed using a radio-TLC scanner.

### Animal studies

All animal experiments were performed in accordance with the National Institutes of Health Guide for the Care and Use of Laboratory Animals, and were approved by the Institutional Animal Care and Use Committee (IACUC) of Samsung Medical Center.

### Biodistribution study

ICR mice (male, 7-week-old, four mice per time point) were injected with [^18^F]**2** (1.85 MBq) dissolved in 10% ethanol–saline via a tail vein. Mice were sacrificed at the indicated time points (2, 30, and 60 min). Samples of blood and major tissues (heart, lung, liver, spleen, kidney, intestines, muscle, femur, and brain) were removed, weighed, and counted in a Wizard^2^ automatic gamma counter (PerkinElmer, Waltham, MA, USA). Data are expressed as the percent injected dose per gram of tissue (% ID/g).

### Optical imaging

Ligand **2** was dissolved in 10% ethanol–10% Cremophor–saline and injected intravenously through a tail vein into 6-week-old Balb/C nude mice (male, 2 mg/kg), which had been fed an alfalfa-free diet for four days prior to this experiment. Optical images were acquired for 1 sec at 2, 30, and 60 min after injection using a Xenogen IVIS Spectrum (PerkinElmer, Waltham, MA, USA) (excitation, 570 nm; emission, 660 nm). After *in vivo* imaging, mice were sacrificed, and tissues of interest were excised and subjected to optical imaging (1-sec exposure). Data are expressed as photons/s/cm^2^/sr (sr: steradian).

### Metabolism study

[^18^F]**2** (19.6 MBq) dissolved in 10% ethanol–saline was injected into ICR mice via a tail vein. At 2, 30, and 60 min after injection, mice were sacrificed, and samples of brain and blood were obtained. The samples were homogenized in 1 mL of acetonitrile and centrifuged. The supernatants were analyzed by radio-TLC using a 4:1 mixture of ethyl acetate–hexane or a 1:1:0.01 mixture of dichloromethane–methanol–triethylamine as the developing solvents.

After *ex vivo* optical imaging, the brain tissues were homogenized in 1 mL of acetonitrile and centrifuged. The supernatants were analyzed by TLC using a 4:1 mixture of ethyl acetate–hexane as the developing solvents, and the TLC plates were subjected to optical imaging for 1 sec (excitation, 570 nm; emission, 660 nm).

## Supplementary information

Supplementary Information
