# Supplementary material for: Synthesis and in vivo characterization of 18F-labeled difluoroboron-curcumin derivative for β-amyloid plaque imaging
Source: Sci Rep. 2019 May 1;9:6747. doi: 10.1038/s41598-019-43257-9 (PMC6494845; doi:10.1038/s41598-019-43257-9)
Supplement: Supplementary file 1 — Supplementary Information [file 41598_2019_43257_MOESM1_ESM.pdf]

Supplementary Information

Synthesis and in vivo characterization of ^{18}F-labeled difluoroboron-curcumin derivative for β-amyloid plaque imaging

Hyunjung Kim[1], Young Hoon Im[2], Jinhee Ahn[2], Jehoon Yang[3], Joon Young Choi[2], Kyung-Han Lee[1,2], Byung-Tae Kim[2] & Yearn Seong Choe[1,2]

[1]Department of Health Sciences and Technology, SAIHST, Sungkyunkwan University, Seoul 06351, Korea
[2]Department of Nuclear Medicine, Samsung Medical Center, Sungkyunkwan University School of Medicine, Seoul 06351, Korea
[3]Laboratory Animal Research Center, Samsung Medical Center, Seoul 06351, Korea

Contents:

^{1}H NMR spectra of ligands (**1-4**)
^{19}F NMR spectra of ligands (**1-4**)
HPLC chromatograms of ligands (**1-4**)
HPLC chromatogram of radioligand ([^{18}F]**2**)
HPLC chromatogram of a mixture of [^{18}F]**2** and **2**
Excitation and emission spectra of **2**
Saturation binding curve of ligand **2** to Aβ aggregates
Proposed polar product standards

^{1}H and ^{19}F NMR spectra of ligands

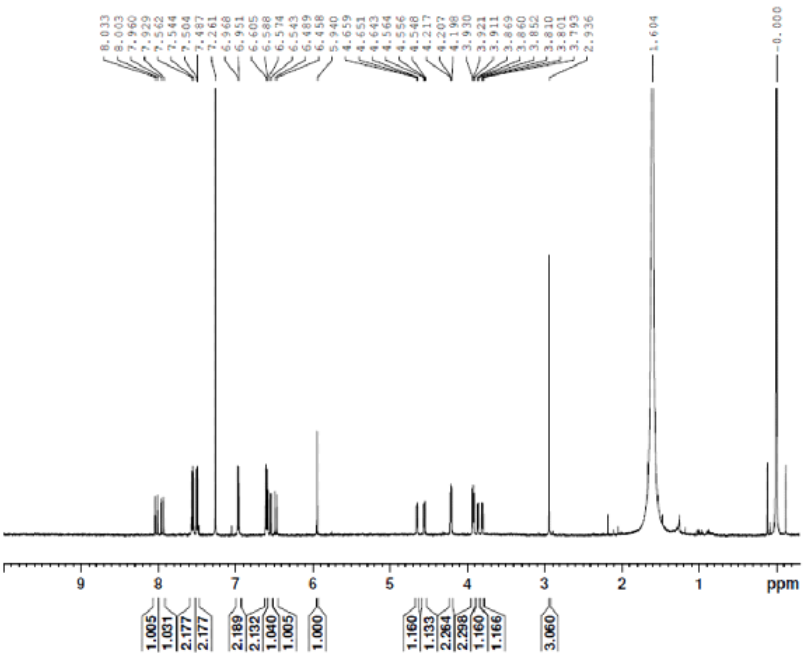

Fig. S1. ^{1}H NMR spectrum of ligand **1**

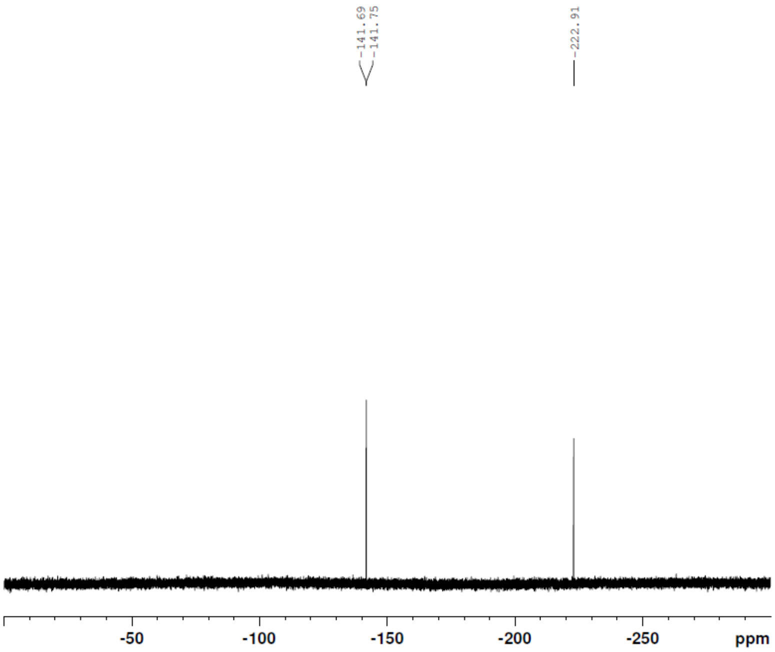

Fig. S2. ^{19}F NMR spectrum of ligand **1**

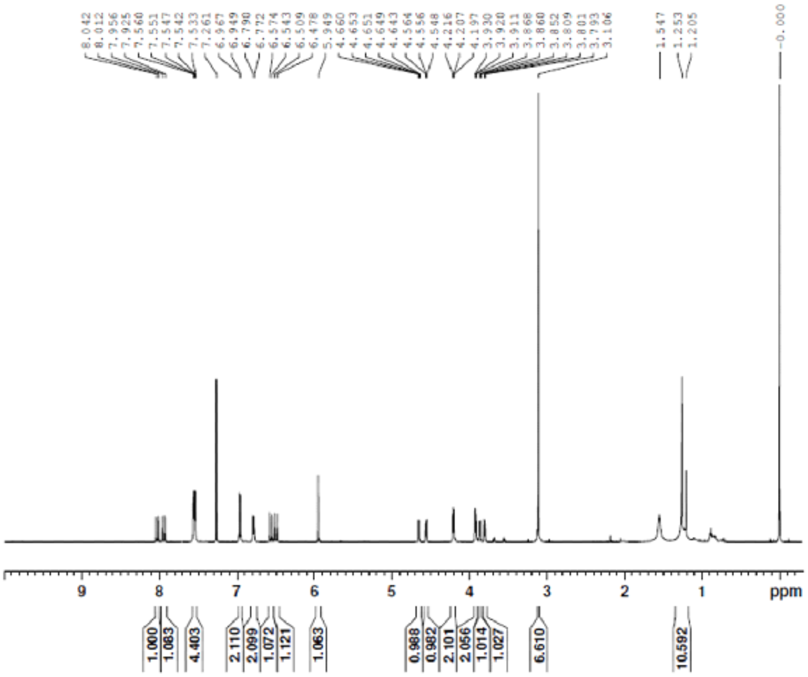

Fig. S3. ^{1}H NMR spectrum of ligand **2**

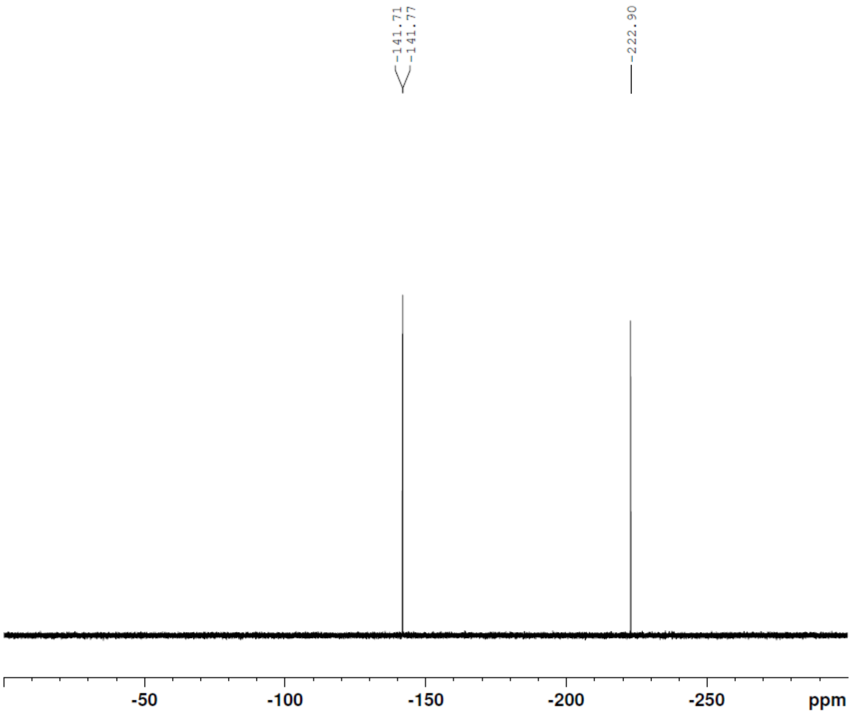

Fig. S4. ^{19}F NMR spectrum of ligand **2**

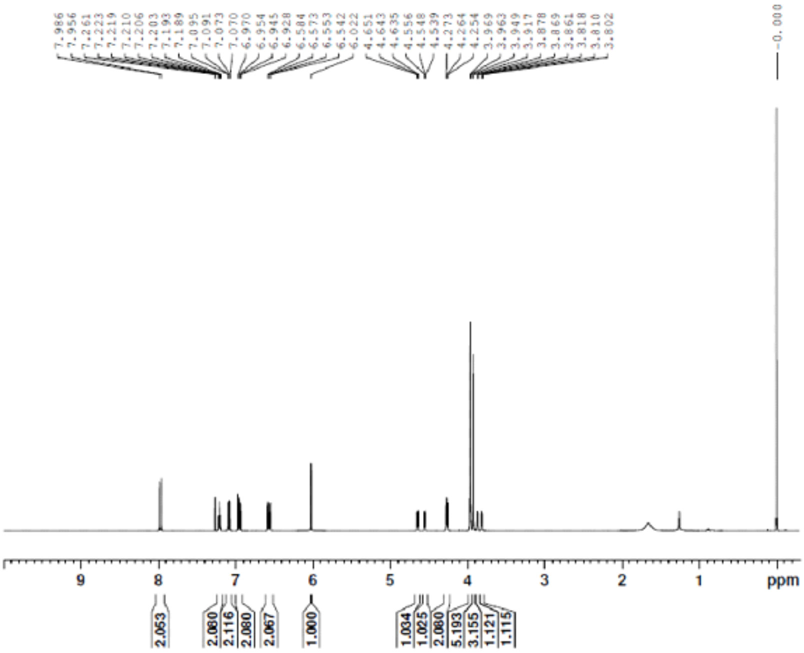

Fig. S5. ^{1}H NMR spectrum of ligand **3**

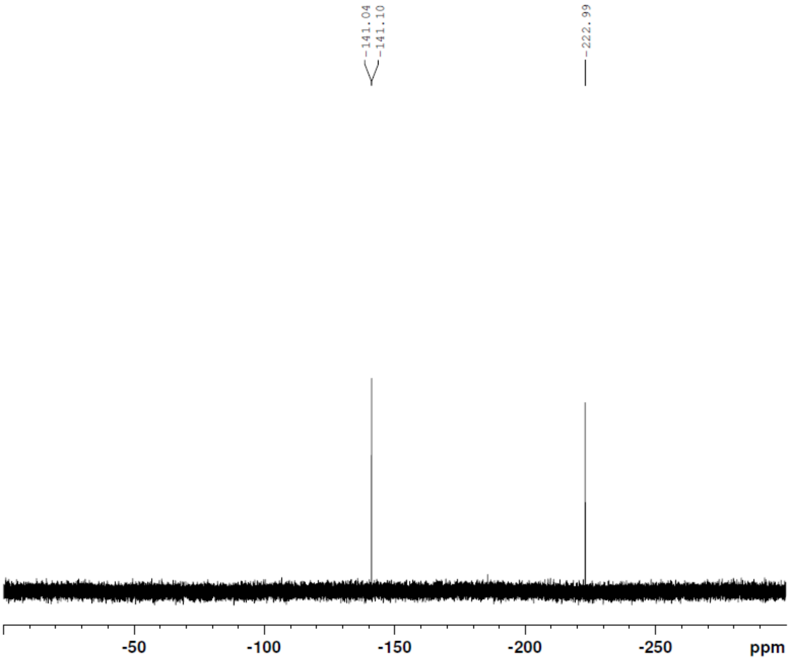

Fig. S6. ^{19}F NMR spectrum of ligand **3**

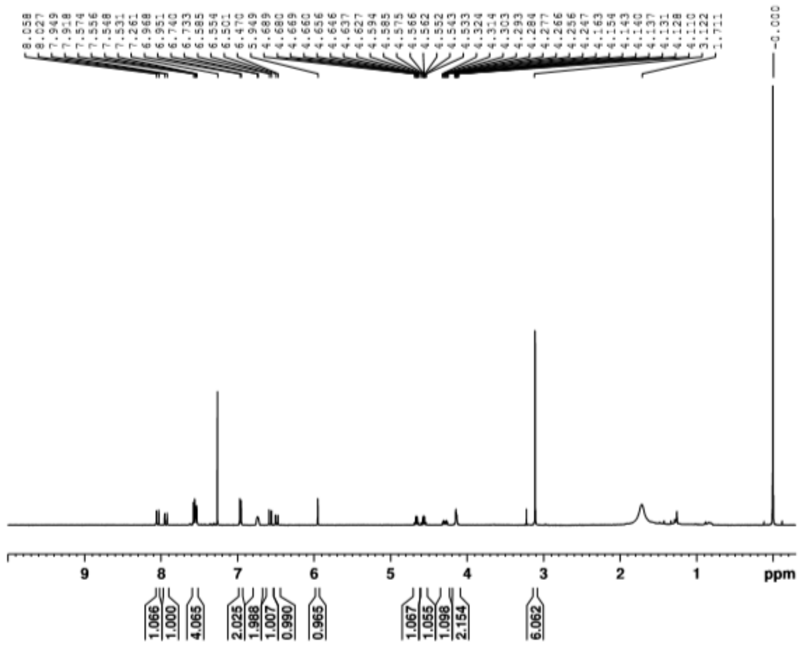

Fig. S7. ^{1}H NMR spectrum of ligand **4**

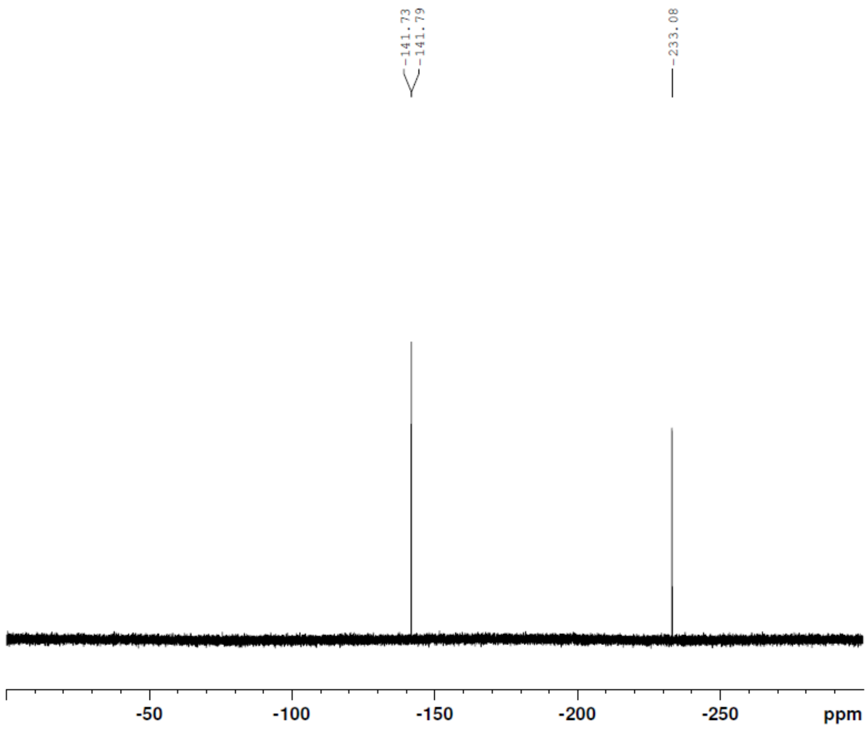

Fig. S8. ^{19}F NMR spectrum of ligand **4**

HPLC chromatograms of non-radioactive ligands

HPLC column: YMC-Pack C18, 4.6 x 250 mm, 5 μm
HPLC solvents: 30:70 TFA (0.1%, aq)-CH_3CN; flow rate: 1 mL/min
Detection: UV (254 nm) detector

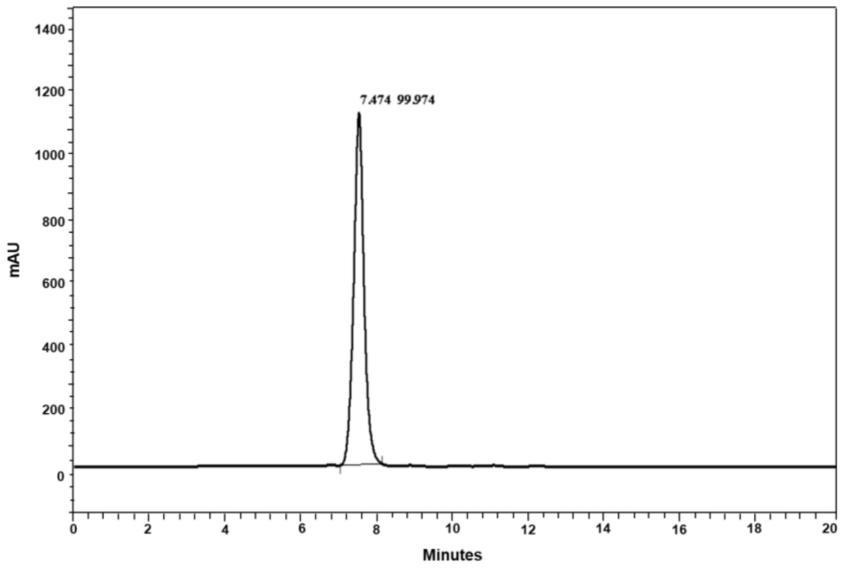

Fig. S9. HPLC chromatogram of ligand **1**
Retention time: 7.474 min
Area % of product: 99.974%

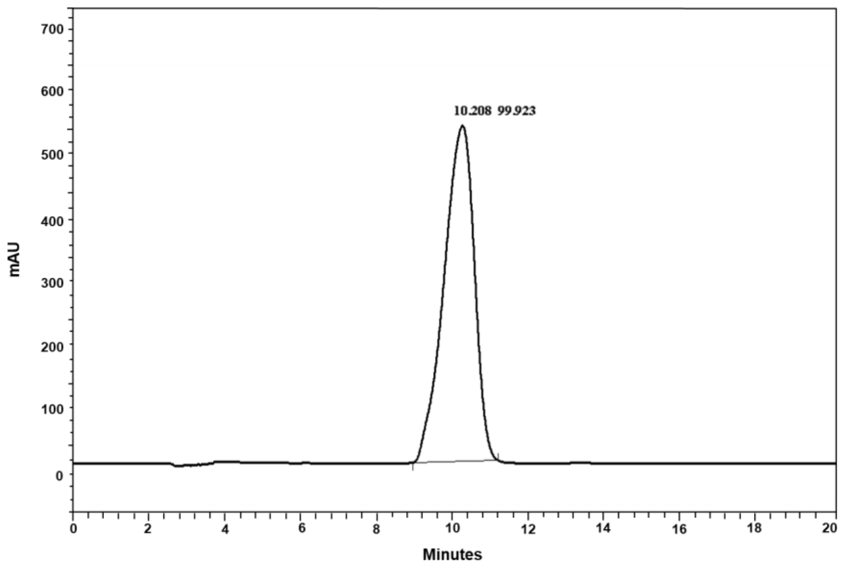

Fig. S10. HPLC chromatogram of ligand **2**
Retention time: 10.208 min
Area % of product: 99.923%

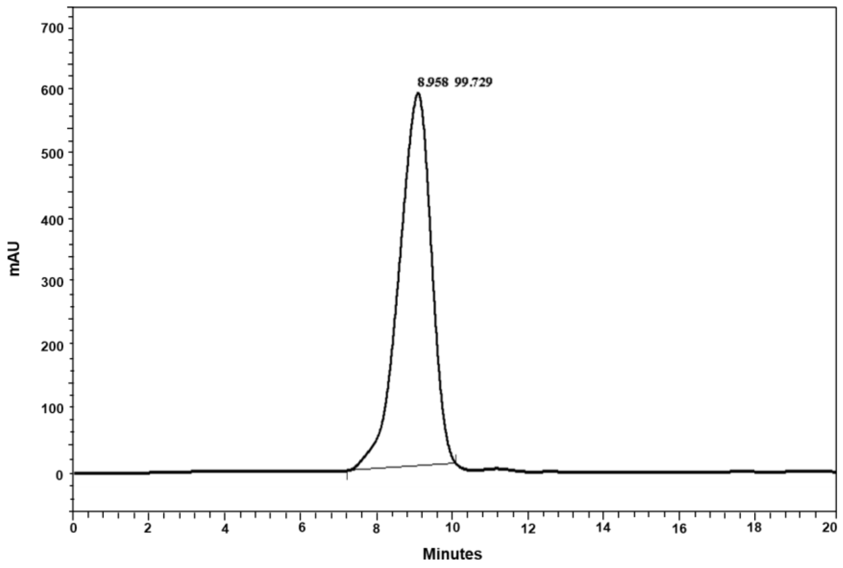

Fig. S11. HPLC chromatogram of ligand **3**
Retention time: 8.958 min
Area % of product: 99.729%

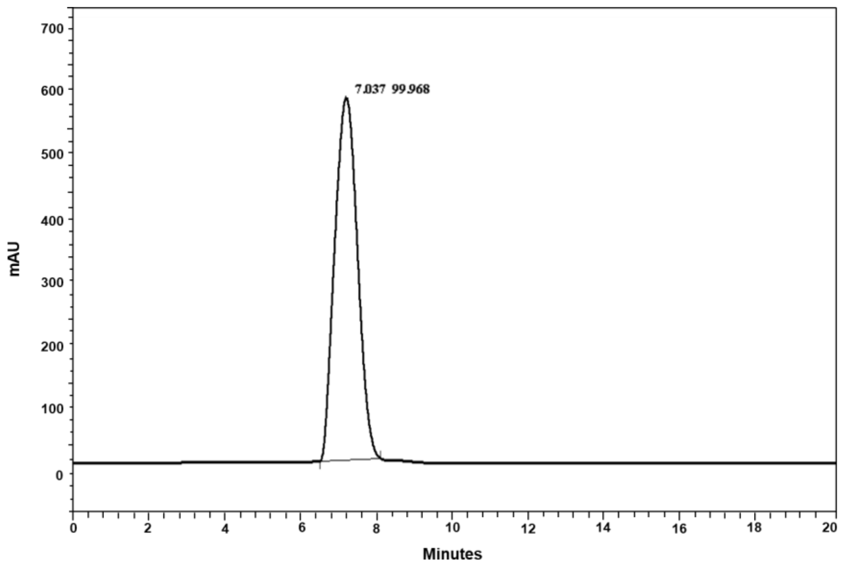

Fig. S12. HPLC chromatogram of ligand **4**
Retention time: 7.037 min
Area % of product: 99.968%

HPLC chromatograms of radioligand

HPLC column: YMC-Pack C18, 4.6 x 250 mm, 5 μm
HPLC solvents: 25:75 TFA (0.1%, aq)-CH_3CN; flow rate: 1 mL/min
Detection: radioactivity detector (red) and UV (254 nm) detector (blue)

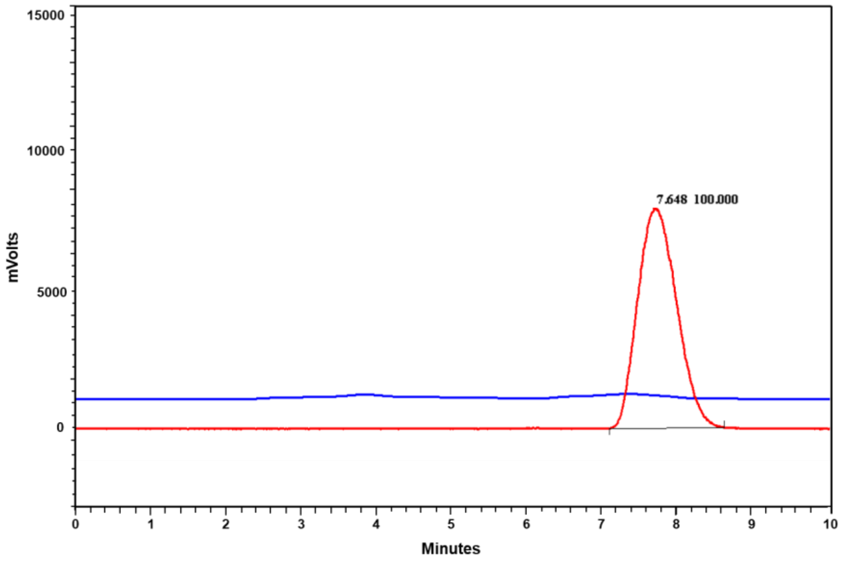

Fig. S13. HPLC chromatogram of ligand [^{18}F]**2**
Retention time: 7.648 min
Area % of product: 100% (radiochemical purity)

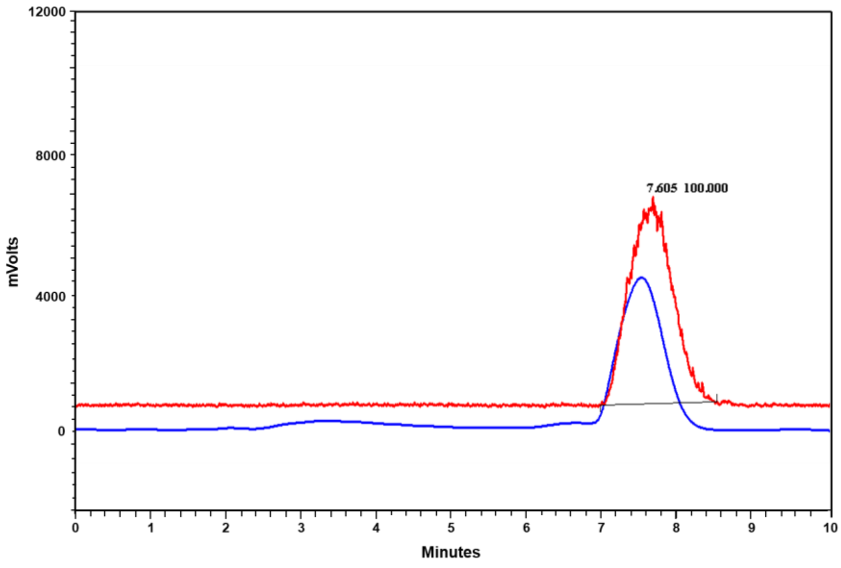

Fig. S14. HPLC chromatogram of a mixture of [^{18}F]**2** and **2**
Retention time: 7.605 min
Area % of product: 100% (radiochemical purity)

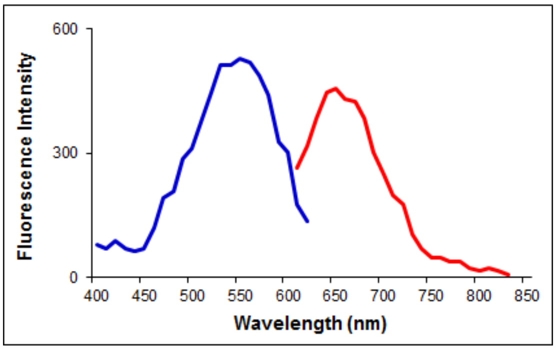

Fig. S15. Excitation and emission spectra of **2** (2.5 μM) in methanol

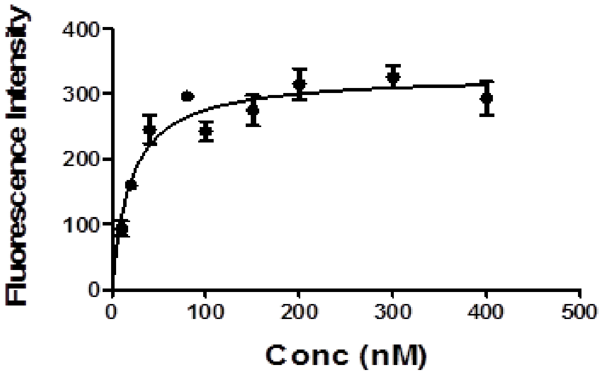

Fig. S16. Saturation binding curve of ligand **2** to Aβ aggregates

Table S1. Proposed polar product standards

Structure	Name	TLC R_f*	TLC R_f**
Polar radioactive products (brain)	–	0	0.59 (major), 0.80, 0.95
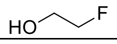	2-Fluoroethanol (Merck)	0.49	0.97
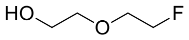	2-(2-Fluoroethoxy)ethanol	–	–
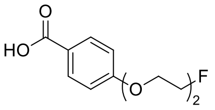	4-(2-(2-Fluoroethoxy)ethoxy) benzoic acid (**S1**)	0.25	0.79
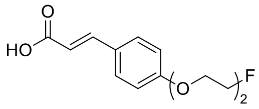	4-(2-(2-Fluoroethoxy)ethoxy) cinnamic acid (**S2**)	0.23	0.76

*TLC plates were developed in a 4:1 mixture of ethyl acetate–hexane and visualized using $KMnO_4$ staining solution.

**TLC plates were developed in a 1:1:0.01 dichloromethane–methanol–triethylamine and visualized using $KMnO_4$ staining solution.

Synthesis of S1 and S2

4-(2-(2-Fluoroethoxy)ethoxy)benzoic acid (S1). 4-(2-(2-Fluoroethoxy)ethoxy)benzaldehyde (15 mg, 0.07 mmol) was dissolved in 0.3 mL of acetone, and to this solution was added dropwise a solution of $KMnO_4$ (16.8 mg, 0.11 mmol) in 0.3 mL of water. The reaction mixture was stirred at rt for 20 min. After the mixture was acidified with 0.1 N HCl to pH 2-3, it was extracted with ethyl acetate, washed with water, and then dried over Na_2SO_4. Flash column chromatography (9.5:0.5 dichloromethane-methanol) gave **S1** (12 mg, 75%) as a white solid. ^{1}H NMR (($CD_3)_2CO$) δ 10.93 (s,1H), 8.00 (d, J = 9 Hz, 2H), 7.07 (d, J =9 Hz, 2H), 4.62 (dt, J = 48 and 2.5 Hz, 2H), 4.27 (t, J = 4.5 Hz, 2H), 3.90 (t, J = 4.5 Hz, 2H), 3.84 (dt, J = 30 and 2.5 Hz, 2H); ^{19}F NMR (($CD_3)_2CO$) δ -223.50; MS (FAB) m/z 229 $(M+H)^+$: HRMS calcd for $C_{11}H_{14}FO_4$, 229.0876; found, 229.0871.

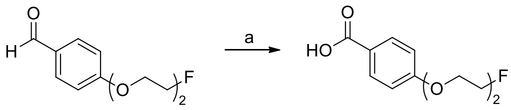

S1

Figure S1. Synthesis of **S1**. Reagents and conditions: (a) $KMnO_4$, acetone-water, rt, 20 min

4-(2-(2-Fluoroethoxy)ethoxy)cinnamic acid (S2). *(E)-Methyl 3-(4-(2-(2-hydroxyethoxy)ethoxy)phenyl)acrylate (**1**).* Methyl 4-hydroxycinnamate (500 mg, 2.81 mmol) and K_2CO_3 (582 mg, 4.21 mmol) were dissolved in 10 mL of DMF, and the solution was stirred at rt for 15 min. After addition of 2-(2-chloroethoxy)ethanol (0.59 mL, 5.61 mmol), the reaction mixture was stirred at 100 ^oC overnight. The mixture was extracted with ethyl acetate, washed with water, saturated NH_4Cl solution, and then dried over Na_2SO_4. Flash column chromatography (1:1 hexane-ethyl acetate) gave **1** (600 mg, 80.2%) as a white solid. ^1H NMR ($CDCl_3$) δ 7.67 (d, *J* = 16 Hz, 1H), 7.48 (d, *J* = 9 Hz, 2H), 6.93 (d, *J* = 8.5 Hz, 2H), 6.33 (d, *J* = 16 Hz, 1H), 4.18 (t, *J* = 4.5 Hz, 2H), 3.90 (t, *J* = 3.5 Hz, 2H), 3.79 (s, 3H) 3.77(t, *J* = 4 Hz, 2H), 3.70 (t, *J* = 3.5 Hz, 2H); MS (EI) *m/z* 266 (M^+): HRMS calcd for $C_{14}H_{18}O_5$, 266.1154; found, 266.1154.

*(E)-Methyl 3-(4-(2-(2-(tosyloxy)ethoxy)ethoxy)phenyl)acrylate (**2**).* Compound **1** (300 mg, 1.13 mmol) was dissolved in 2 mL dichloromethane, and to this solution was added *p*-toluenesulfonyl chloride (333 mg, 1.35 mmol). After addition of triethylamine (0.94 mL, 6.76 mmol) at 0 ^oC (ice bath), the reaction mixture was stirred at rt overnight. After the reaction was quenched with saturated NH_4Cl solution, the reaction mixture was extracted with dichloromethane, washed with water, and then dried over Na_2SO_4. Flash column chromatography (1:1 hexane-ethyl acetate) gave **2** (430 mg, 90.8%) as a white solid**.** ^1H NMR ($CDCl_3$) δ 7.80 (d, *J* = 8 Hz, 2H), 7.66 (d, *J* = 16 Hz, 1H), 7.47 (d, *J* = 9 Hz, 2H), 7.31 (d, *J* = 8.5 Hz, 2H), 6.90 (d, *J* = 9 Hz, 2H), 6.33 (d, *J* = 16 Hz, 1H), 4.20 (t, *J* = 5 Hz, 2H), 4.09 (t, *J* = 5 Hz, 2H), 3.81 (t, *J* = 3 Hz, 2H), 3.80 (s, 3H), 3.77 (t, *J* = 5 Hz, 2H), 2.41 (s, 3H); MS (EI) *m/z* 420 (M^+): HRMS calcd for $C_{21}H_{24}O_7S$, 420.1243; found, 420.1241.

(E)-Methyl 3-(4-(2-(2-fluoroethoxy)ethoxy)phenyl)acrylate (3). Compound **2** (98 mg, 0.23 mmol) was dissolved in 5 mL *t*-BuOH, and to this solution was added CsF (106 mg, 0.70 mmol). After the reaction mixture was stirred at 100 ^oC overnight, it was extracted with ethyl acetate, washed with water, and then dried over Na_2SO_4. Flash column chromatography (2:1 hexane-ethyl acetate) gave **3** (48 mg, 77.8%) as a white solid. ^1H NMR ($CDCl_3$) δ 7.66 (d, *J* = 16 Hz, 1H), 7.48 (d, *J* = 9 Hz, 2H), 6.93 (d, *J* = 9 Hz, 2H), 6.33 (d, *J* = 16 Hz, 1H), 4.65 (dt, *J* = 47.5 and 4 Hz, 2H), 4.19 (t, *J* = 5 Hz, 2H), 3.92 (t, *J* = 5 Hz, 2H), 3.86 (dt, *J* = 29.5 and 4 Hz, 2H), 3.80 (s, 3H); MS (EI) *m/z* 268 (M^+): HRMS calcd for $C_{14}H_{17}FO_4$, 268.1111; found, 268.1106.

*(E)-3-(4-(2-(2-Fluoroethoxy)ethoxy)phenyl)acrylic acid (**S2**).* Compound **3** (30 mg, 0.11 mmol) was dissolved in 1 mL MeOH, and to this solution was added dropwise a solution of NaOH (13.4 mg, 0.34 mmol) in 0.5 mL water. The reaction mixture was stirred at rt overnight. After the mixture was acidified with 0.1 N HCl to pH 2-3, it was extracted with ethyl acetate, washed with water, and then dried over Na_2SO_4. Flash column chromatography (9.5:0.5 dichloromethane-methanol) gave **S2** (15 mg, 53.7%) as a white solid. ^1H NMR ($(CD_3)_2CO$) δ 10.60 (s,1H), 7.65 (d, *J* = 9 Hz, 3H), 7.03 (d, *J* =9 Hz, 2H), 6.41 (d, *J* = 16 Hz, 1H), 4.62 (dt, *J* = 47.5 and 3 Hz, 2H), 4.23 (t, *J* = 5 Hz, 2H), 3.89 (t, *J* = 3.5 Hz, 2H), 3.83 (dt, *J* = 30.5 and 3 Hz, 2H); ^{19}F NMR ($(CD_3)_2CO$) δ -223.49; MS (EI) *m/z* 254 (M^+): HRMS calcd for $C_{13}H_{15}FO_4$, 254.0954; found, 254.0950.

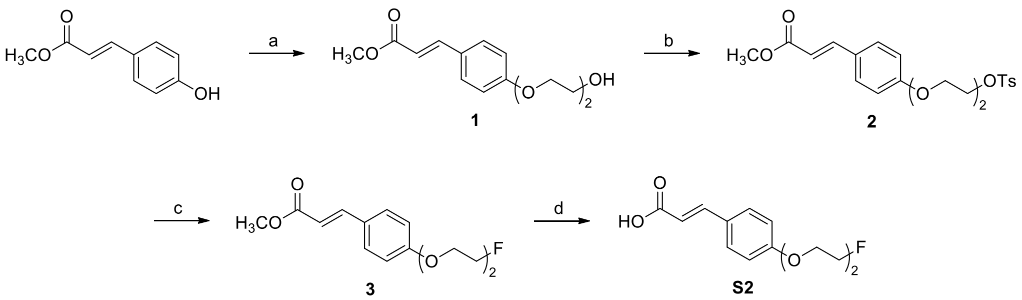

Figure S2. Synthesis of **S2**. Reagents and conditions: (a) 2-(2-chloroethoxy)ethanol, K_2CO_3, DMF, 100 ^oC, overnight; (b) TsCl, Et_3N, CH_2Cl_2, rt, overnight; (c) CsF, *t*-BuOH, 100 ^oC, overnight; (d) NaOH, MeOH-water, rt, overnight
